# Mechanical strain treatment improves nuclear transfer reprogramming efficiency by enhancing chromatin accessibility

**DOI:** 10.1016/j.stemcr.2023.02.007

**Published:** 2023-03-23

**Authors:** Yujie Chen, Ruimin Xu, Shuang Zhou, Chengchen Zhao, Ziyue Hu, Yuwei Hua, Yanhong Xiong, Xiaoyu Liu, Junhong Lü, Yao Sun, Chong Li, Shaorong Gao, Yong Zhang

**Affiliations:** 1 Institute for Regenerative Medicine, Shanghai East Hospital, Shanghai Key Laboratory of Signaling and Disease Research, Frontier Science Center for Stem Cell Research, School of Life Sciences and Technology, Tongji University, Shanghai 200092, China; 2 Department of Implantology, School & Hospital of Stomatology, Tongji University, Shanghai, China; 3 Shanghai Advanced Research Institute, Chinese Academy of Sciences, Shanghai 201203, China; 4 College of Pharmacy, Binzhou Medical University, Yantai 264003, China; 5 Shanghai Key Laboratory of Maternal Fetal Medicine, Shanghai Institute of Maternal-Fetal Medicine and Gynecologic Oncology, Clinical and Translation Research Center, Shanghai First Maternity and Infant Hospital, Frontier Science Center for Stem Cell Research, School of Life Science and Technology, Tongji University, Shanghai 200092, China

**Keywords:** mechanical strain, reprogramming, chromatin accessibility, SCNT, embryonic genome activation

## Abstract

Cellular mechanical properties are considered to be important factors affecting cell fate transitions, but the links between cellular mechanical properties and transition efficiency and chromatin structure remain elusive. Here, we predicted that mechanical strain treatment could induce signatures of cellular dedifferentiation and transdifferentiation, and we validated this prediction by showing that mechanical strain-treated mouse cumulus cells (CCs) exhibit significantly improved somatic cell nuclear transfer (SCNT) reprogramming efficiency. We found that the chromatin accessibility of CCs was globally increased by mechanical strain treatment and that this increase was partially mediated by the induction of the YAP-TEAD interaction. Moreover, using mechanical strain-treated CCs could prevent transcriptional dysregulation in SCNT embryos. Taken together, our study results demonstrated that modulating cell mechanical properties to regulate epigenetic status is a promising approach to facilitate cell fate transition.

## Highlights

• Modulation of mechanical properties can facilitate efficient SCNT
• Mechanically enhanced chromatin remodeling affects cell fate reprogramming potential
• ms-SCNT embryos partially repaired dysregulated genes in EGA
• An integrative approach to evaluate the consequences of mechanical-induced transition

## Graphical abstract

**Figure:**
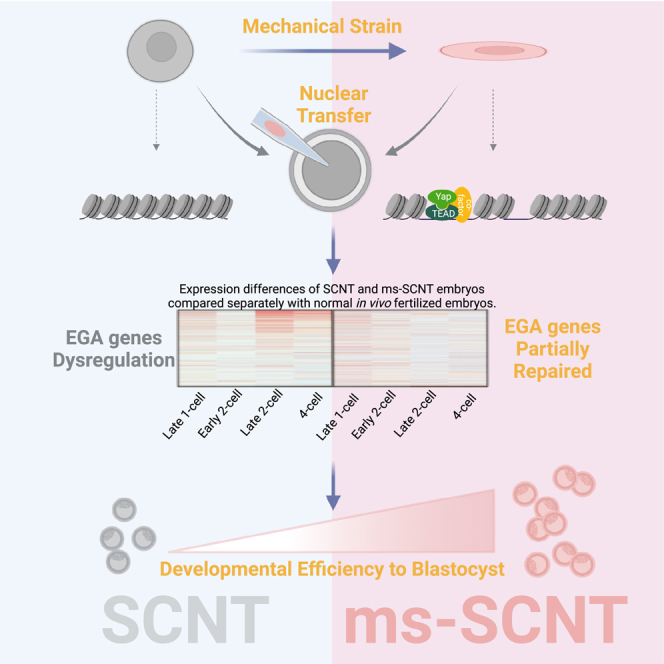

## Teaser

Chen et al. provide an integrative approach to address the question of how to clarify the relationship between the quantifiable modulation of mechanical properties and cell fate transitions, including mechanical manipulation, rapid examination of transition consequences, and high-throughput epigenetic evaluation. They first report that modulation of an appropriate extracellular force can dramatically increase chromatin accessibility and effectively facilitate somatic cell nuclear transfer (SCNT), indicating that chromatin remodeling plays a mediating role in linking the response to mechanical treatment and cell fate reprogramming potential.

## Introduction

Recently, cellular mechanical properties have been demonstrated to play important roles in multiple cell fate determination events, including stem cell lineage commitment (Engler et al., 2006; McBeath et al., 2004), differentiation or self-renewal induction (Chowdhury et al., 2010; Connelly et al., 2010; Gilbert et al., 2010), and cell allocation patterns during development (Chan et al., 2019). Although extracellular forces could be transmitted to the nucleus interior and lead to chromatin stretching and the expression of reporter genes (Tajik et al., 2016), applying cellular mechanical properties to induce cell fate transitions is still challenging, mainly because identifying the appropriate extracellular force to effectively facilitate cell fate transition is a time-consuming and labor-intensive process.

We aimed to overcome this challenge by considering the following two issues. First, we relied on previously collected time-series gene expression data during cell state transitions and defined gene signatures for those processes (Zhu et al., 2017). These gene signatures of cell state transitions could be used to evaluate the directed reprogramming potential of cells upon extracellular force treatments by comparing their differentially expressed genes, and this evaluation procedure can accelerate the fine-tuning of the effective extracellular force. Second, somatic cell nuclear transfer (SCNT) is an ideal system in which to validate the extracellular force-induced reprogramming for mouse cells, as the measurement of the effectiveness is quick and clear, i.e., the rate of blastocyst formation at day 3.5 post nuclear transfer. It has been reported that SCNT reprogramming efficiency can be improved by altering the epigenetic status of donor nuclei (Matoba and Zhang, 2018). However, to the best of our knowledge, there has been no report on the use of mechanical property modulation to improve SCNT reprogramming efficiency. In this study, we applied an integrative approach to predict that mechanical strain treatment could induce signatures of cellular dedifferentiation and transdifferentiation and validated this prediction by showing that mechanical strain-treated mouse somatic cells exhibit significantly improved SCNT reprogramming efficiency, which demonstrated that modulating mechanical properties to regulate epigenetic status is a promising approach to achieve cell fate transition.

## Results

### Mechanical strain treatment improved SCNT efficiency

**Figure 1:**
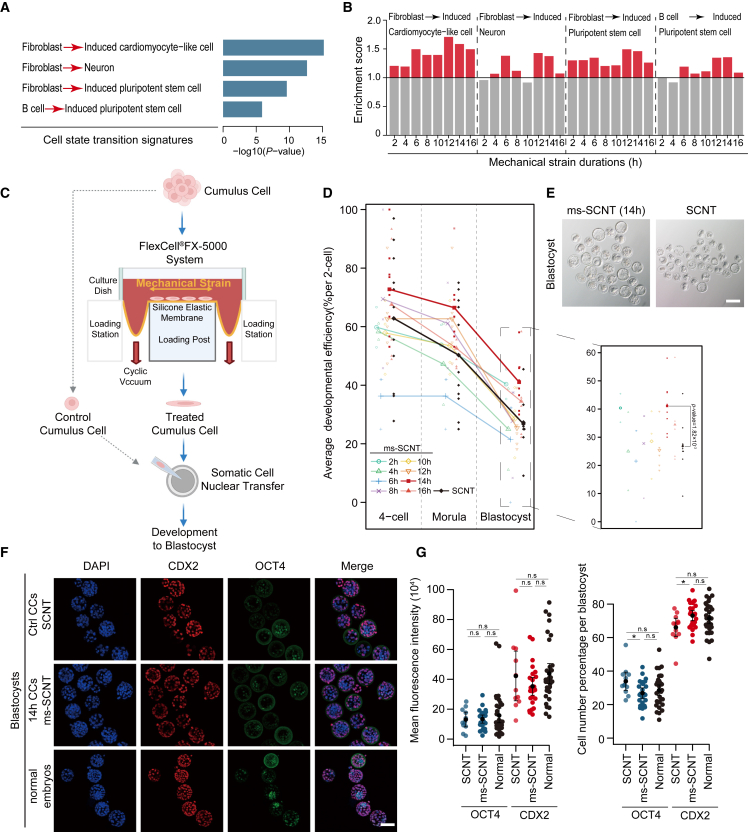
Mechanical strain treatment improved SCNT efficiency (A) The bar plot demonstrates the top four cell state transition signatures identified by CSTEA. (B) The bar plot demonstrates the signature enrichment in each duration, and those with an enrichment score <1 (gray) were considered not enriched. (C) The ms-SCNT embryo procedure, created with BioRender (https://app.biorender.com). (D) The developmental efficiency of ms-SCNT embryos across different mechanical strain durations and SCNT embryos (n ≥ 2). (E) The blastocyst phenotype of 14 h ms-SCNT and SCNT embryos. Scale bar, 100 μm. (F) CDX2 and OCT4 staining in 14 h ms-SCNT, SCNT, and normal *in vitro* fertilization blastocysts (n = 3). Scale bar, 100 μm. (G) Scatterplot demonstrating the quantification of positive CDX2 and OCT4 staining (left), and the percentage of positive cells in each blastocyst (right). Significant differences, ^∗^p < 0.05.

To investigate whether mechanical strain treatment can facilitate cell fate transition, we exposed mouse cumulus cells (CCs), which are common SCNT donors, to a biaxial cyclic mechanical strain of 7.5% at 0.5 Hz for durations ranging from 2 to 16 h, with 2 h intervals, and performed RNA sequencing (RNA-seq) for each condition (Figure S1A; Table S1). Since over-expressing reprogramming factors can facilitate cell fate transitions, and since activators may play central roles in reconstructing transcriptional networks (Graf and Enver, 2009), we focused on differentially upregulated genes to assess the cellular reprogramming potentials. Differentially upregulated genes between samples of adjacent durations were grouped, and the cell state transition signature analysis (Zhu et al., 2017) revealed that these genes were highly enriched in the signatures of cellular dedifferentiation and transdifferentiation (Figure 1A; see experimental procedures for details). For each mechanical strain duration, we identified differentially upregulated genes between mechanical strain-treated and control CCs (gene numbers: 760 for 2 h; 857 for 4 h; 651 for 6 h; 970 for 8 h; 661 for 10 h; 521 for 12 h; 541 for 14 h; 607 for 16 h), which we regarded as mechanical strain-induced genes, and calculated their enrichment scores to identify highly enriched cell state transition signatures (see experimental procedures for details). The 12 and 14 h mechanical strain durations showed the highest enrichment scores in all signatures of cellular dedifferentiation and transdifferentiation (Figure 1B), suggesting that appropriate mechanical strain treatment might enhance the cellular reprogramming potential of CCs.

To confirm the enhancement of cellular reprogramming or transdifferentiation potential of mechanical strain-treated CCs, we performed SCNT using CCs exposed to mechanical strain for different durations as donors and obtained mechanical strain-treated SCNT (ms-SCNT) embryos (Figure 1C). The ms-SCNT embryos from several duration groups exhibited significantly higher rates of blastocyst formation than control SCNT embryos (26.8%), with the 14 h duration showing the highest rate (41.6%) (Figures 1D and 1E), consistent with the 14 h duration displaying one of the highest enrichment scores in terms of cellular dedifferentiation and transdifferentiation signatures. In addition to CCs, ms-SCNT embryos generated from 14 h mechanical strain-treated mouse embryonic fibroblast cells and tail-tip fibroblast cells displayed higher (statistically not significant) blastocyst formation rates (embryonic fibroblast cells: 25.40% for control, 40.14% for 14 h mechanical strain treatment; tail-tip fibroblast cells: 28.68% for control, 34.35% for 14 h mechanical strain treatment; Figures S1B–S1E), confirming the effectiveness of mechanical strain treatment for improving SCNT efficiency for other cell types. We next focused on ms-SCNT embryos based on CCs treated with mechanical strain for 14 h. We labeled inner cell mass (ICM) and trophectoderm (TE) cells with OCT4 and CDX2, respectively, and the immunofluorescence intensities of both markers showed no significant differences between ms-SCNT and normal blastocysts (Figures 1F and 1G), confirming the quality of the ms-SCNT blastocysts. To evaluate whether mechanical strain treatment can cause DNA damage, we performed γH2AX immunofluorescence staining for 14 h mechanical strain-treated, etoposide-treated, and control CCs, and no statistical differences were observed between mechanical strain-treated and control CCs (Figures S1F and S1G), suggesting that 14 h mechanical strain treatment may not cause DNA damage. Our results demonstrated the effectiveness of mechanical strain treatment on improving SCNT efficiency, at least up to the blastocyst stage.

### Mechanical strain treatment increased chromatin accessibility in CCs

**Figure 2:**
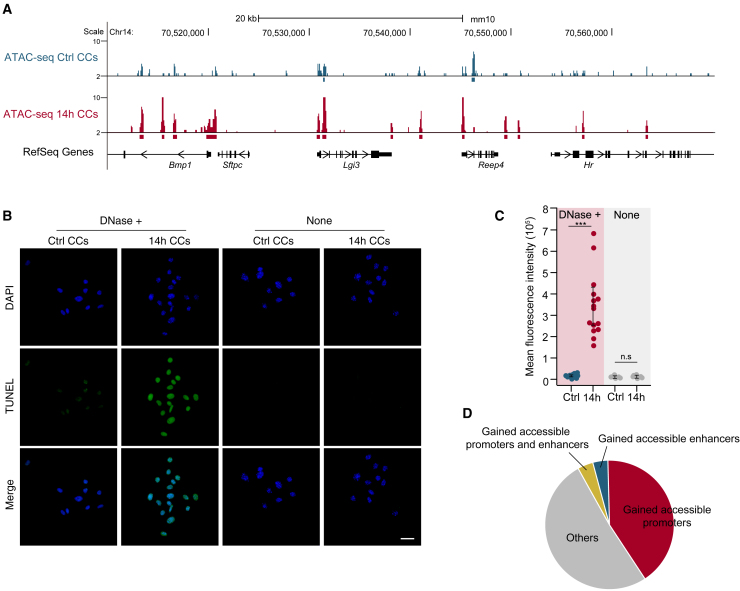
Mechanical strain treatment increased the chromatin accessibility of CCs (A) Genome browser view of a representative region as an example to illustrate that the ATAC-seq signals increased at some promoter regions after 14 h of mechanical strain exposure (n = 2). (B) TUNEL assay of the control and 14 h mechanical strain-treated CCs with or without DNase treatment (n = 3). Scale bar, 20 μm. (C) Quantification of positive signals in the TUNEL assay with or without DNase treatment. ^∗∗∗^p < 0.001. (D) The pie chart demonstrates the percentage of upregulated genes associated with regions of chromatin accessibility gain in the 14 h mechanical strain-induced cells.

We next investigated the effects of 14 h mechanical strain treatment on CCs. As chromatin accessibility is closely related to gene transcription regulation and is sensitive to extracellular mechanical environments (Stowers et al., 2019), we suspected that mechanical strain treatment might alter the chromatin accessibility of the CCs. To test this assumption, we performed assay for transposase-accessible chromatin (ATAC)-seq in mechanical strain-treated and control CCs (Table S1). We observed a global increase in chromatin accessibility in mechanical strain-treated CCs (Figure 2A), with a large number of newly accessible chromatin regions gained upon mechanical strain treatment (38,228 and 92,419 accessible chromatin regions in control and mechanical strain-treated CCs, respectively). To confirm the global increase in chromatin accessibility upon mechanical strain treatment, we performed a DNase-TUNEL assay in mechanical strain-treated and control CCs. The fluorescence signals were much stronger in mechanical strain-treated CCs than in the control cells (Figures 2B and 2C), consistent with the ATAC-seq profiles. A total of 15.4% and 29.4% of gained accessible chromatin regions were located in promoters and potential enhancers (see experimental procedures for details), 48.8% of mechanical strain-induced genes gained accessible promoters or potential enhancers, and those genes were functionally enriched in differentiation and developmental processes (Figures 2D and S2A). Our results demonstrated that mechanical strain treatment globally increased the chromatin accessibility of CCs, with functional implications for reprogramming potential.

As the quality of SCNT embryos is enhanced when donor cells are maintained in the G0/G1 phase (Wakayama et al., 1998), we examined the cell-cycle distributions of mechanical strain-treated CCs. No significant differences were observed between mechanical strain-treated and control CCs (Figure S2B), excluding the possibility that mechanical strain treatment increased SCNT efficiency by altering the cell cycle in CCs. Because H3K9me3 has been reported to be an epigenetic barrier to SCNT reprogramming (Liu et al., 2016; Matoba et al., 2014), we further investigated the H3K9me3 profiles of mechanical strain-treated and control CCs by performing H3K9me3 chromatin immunoprecipitation (ChIP)-seq (Table S1). The percentages of H3K9me3-enriched loci were similar between mechanical strain-treated and control CCs (2.34% and 2.68%, respectively), consistent with the comparable H3K9me3 immunofluorescence staining in the two samples (Figures S2C and S2D), indicating that H3K9me3 signals were not dramatically reduced by mechanical strain treatment.

### YAP contributed to the mechanical strain-induced increase in chromatin accessibility in CCs

**Figure 3:**
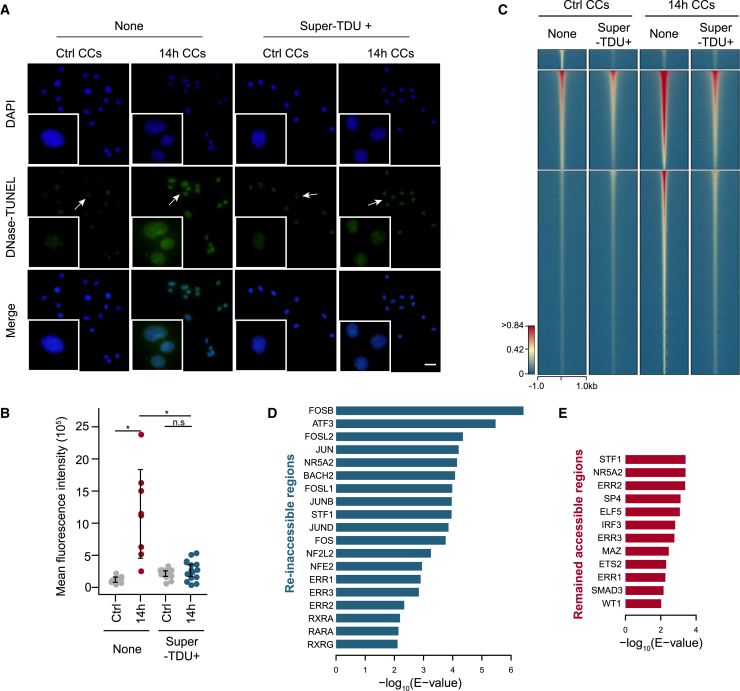
YAP contributed to the mechanical strain-induced chromatin accessibility gain of CCs (A) DNase-TUNEL assay of the control and 14 h mechanical strain-treated CCs with or without super-TDU (n = 3). Scale bar, 20 μm. (B) The quantification of positive TUNEL signals in samples with or without DNase treatment. ^∗^p < 0.05. (C) Heatmap of normalized ATAC-seq signals around peak summits demonstrated a genome-wide increase in chromatin accessibility induced by mechanical strain, while ATAC-seq signals decreased upon super-TDU treatment (n = 2). Heatmap clustering was ordered from strongest to weakest signal. (D and E) Bar plot showing the significance of *de novo* motif discovery in reinaccessible regions (D) and remained-accessible regions (E).

Since YAP is known to modulate transcription in response to mechanical signals (Yu and Guan, 2013), and since TEAD interacts with YAP to mediate YAP function (Zhao et al., 2008), probably by cooperating with AP-1 (Stein et al., 2015), we next investigated whether YAP is responsible for the increase in chromatin accessibility in mechanical strain-treated CCs. We added Super-TDU, a competitive inhibitor of the YAP-TEAD interaction (Jiao et al., 2014), to CCs prior to the mechanical strain treatment to dissect the chromatin function of YAP mediated by TEAD (Figure S3A). The DNase-TUNEL assay revealed significantly weaker fluorescence signals when Super-TDU was added than when CCs were treated with mechanical strain alone (Figures 3A and 3B). We further performed ATAC-seq in Super-TDU-supplemented mechanical strain-treated and control CCs (Table S1). While the addition of Super-TDU did not strongly influence the number of accessible chromatin regions in control CCs (38,228 and 39,376 without and with Super-TDU added, respectively), the ATAC-seq signals at accessible regions were slightly attenuated upon Super-TDU addition (Figure S3B). The number of accessible chromatin regions in mechanical strain-treated CCs dramatically decreased upon Super-TDU addition (92,419 and 68,394 without and with Super-TDU added, respectively). For mechanical strain-treated CCs, the decrease in accessible chromatin regions upon Super-TDU treatment mainly occurred at the gained accessible chromatin regions (Figures 3C, S3C, and S3D). We further divided the gained accessible chromatin regions into those that reverted to inaccessibility (“reinaccessible”) and those that remained accessible upon Super-TDU treatment and performed motif analysis on both groups (see experimental procedures for details). The motifs of AP-1 factors (FOSB, FOSL2, JUN, FOSL1, JUNB, JUND, and FOS) were highly enriched in the group of reinaccessible regions (Figures 3D and S3E; Table S2), suggesting the involvement of YAP in the establishment and maintenance of chromatin accessibility in those regions, as the AP-1 motif was reported to be enriched in YAP ChIP-seq peaks (Stein et al., 2015). The motifs of nuclear receptors (STF1, NR5A2, and ERR2) showed the highest enrichment in the group of remained-accessible regions (Figure 3E). Our results indicated that the YAP-TEAD interaction is responsible for the establishment of approximately half of the chromatin regions that gained accessibility upon mechanical strain treatment, while additional factors may contribute to the remaining regions.

### ms-SCNT embryos rescued the dysregulation of genome activation

**Figure 4:**
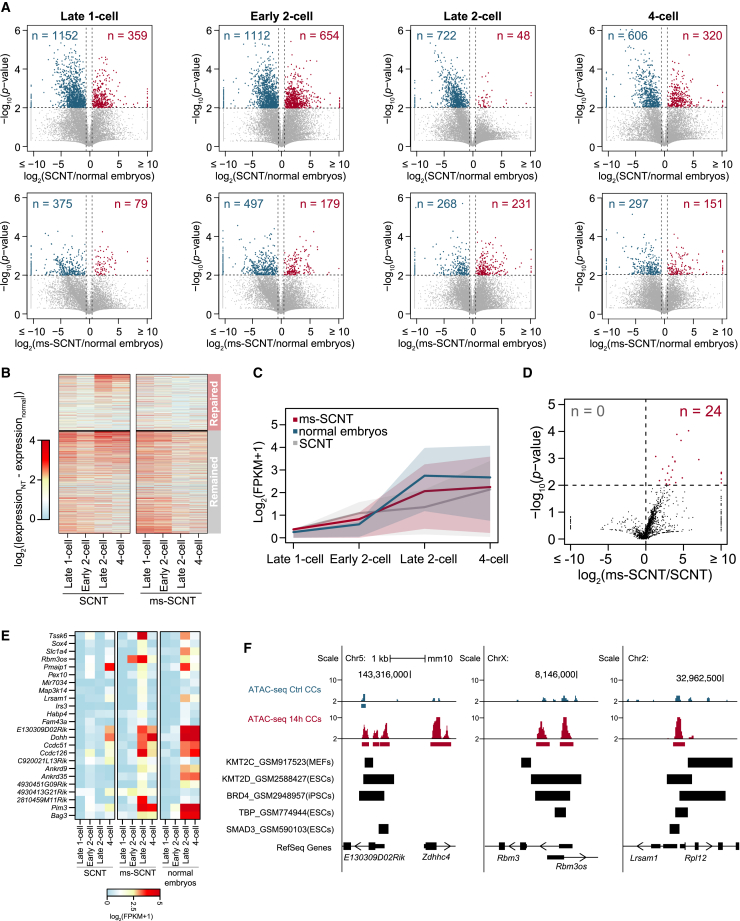
Genome activation dysregulation is repaired in ms-SCNT embryos (A) Volcano plot of gene expression levels compared between SCNT and normal *in vivo* fertilized embryos (top) and between ms-SCNT and normal *in vivo* fertilized embryos (bottom) at the late 1-cell, early 2-cell, late 2-cell, and 4-cell stages (n ≥ 2). Significantly differentially expressed genes are highlighted in blue and red. (B) The heatmap demonstrates the expression level differences between normal *in vivo* fertilized embryos in SCNT and ms-SCNT embryos for the 2,865 genes downregulated in SCNT embryos. (C) The line plot shows the expression level of nonmaternally loaded EGA genes in SCNT, ms-SCNT, and normal embryos during early development. (D) Volcano plot of gene expression levels of nonmaternally loaded EGA genes compared between SCNT and ms-SCNT embryos at the late 2-cell stage. (E) Heatmap displaying the expression levels of 24 nonmaternally loaded EGA genes. (F) Genome browser snapshots of representative promoter regions of nonmaternally loaded EGA genes. The tracks include the chromatin regulator binding sites predicted by Cistrome DB Toolkit significance analysis.

We next investigated the transcriptomes of ms-SCNT embryos to understand the mechanisms underlying the improvement in efficiency. Because embryonic genome activation (EGA), the major phase of which occurs at the late 2-cell stage, is critical for the development of SCNT embryos (Matoba and Zhang, 2018), we performed RNA-seq at four stages covering the whole process of EGA (i.e., late 1 cell, early 2 cell, late 2 cell, and 4 cell) in SCNT and ms-SCNT embryos (Table S1). Compared with normal embryos, SCNT embryos exhibited 2,865 significantly downregulated genes across the EGA process, whereas only 1,142 genes were upregulated (Figure 4A), indicating that downregulation is the major type of transcriptional dysregulation in SCNT embryos. In contrast to SCNT embryos, ms-SCNT embryos showed a much smaller difference in transcriptional profile than normal embryos, with 1,298 and 598 down- and upregulated genes, respectively (Figure 4A; Table S3), suggesting the compensation of transcriptional dysregulation in ms-SCNT embryos, especially for those genes that were downregulated in SCNT embryos. Among the 2,865 genes downregulated in SCNT embryos, 35.5% displayed repaired transcription levels in ms-SCNT embryos (Figure 4B), and those repaired downregulated genes were functionally enriched in cell-cycle and blastocyst formation (Figure S4A). Our results suggested that using mechanical strain-treated CCs could partially repair the transcriptional dysregulation in SCNT embryos.

To further investigate the improvement of EGA in ms-SCNT embryos, we focused on the transcriptional patterns of 1,946 nonmaternally loaded EGA genes (see experimental procedures for details). These genes displayed a clear trend of transcription-level elevation from the late 2-cell stage in normal embryos (Figure 4C; Table S4). The transcription patterns of those EGA genes in normal embryos were more similar to those in ms-SCNT embryos than those in SCNT embryos (Figures 4C and S4B), suggesting that ms-SCNT embryos repaired the dysregulation of some EGA genes. 24 EGA genes were significantly upregulated at the late 2-cell stage in ms-SCNT embryos compared with SCNT embryos, while none of the EGA genes were significantly downregulated (Figure 4D); moreover, those 24 genes displayed similar expression patterns between ms-SCNT embryos and normal embryos (Figure 4E). Those transcriptional activation-repaired EGA genes included *Pim3*, *Pex10*, and *Sox4*, which were reported to be important for pluripotency or embryo development (Aksoy et al., 2007; Bhattaram et al., 2010; Hanson et al., 2014; Schilham et al., 1996), suggesting that the compensation of the dysregulation of some EGA genes might contribute to the improved efficiency of ms-SCNT embryos. Among the 24 genes, 8 gained accessible chromatin regions around their transcription start sites (TSSs) in CCs (Figures 4F and S4C). We further predicted that chromatin regulators including KMT2C/D, BRD4, SMAD3, and TBP have the potential to bind accessible chromatin regions around their TSSs (see experimental procedures for details); these regulators have also been reported to be functionally important during reprogramming or pluripotency maintenance (Di Micco et al., 2014; Dunn et al., 2004; Veenstra et al., 2000; Wang et al., 2016) (Figures 4F and S4D), suggesting that mechanical strain treatment might contribute to the binding of reprogramming factors in ms-SCNT embryos to repair the dysregulation of genome activation.

## Discussion

This study utilized mechanical strain to improve SCNT reprogramming efficiency, which emphasized the relationship between the quantifiable modulation of mechanical properties and cell fate transition. We further demonstrated that the improvement was achieved via a mechanical strain-induced increase in chromatin accessibility, which indicated that chromatin remodeling can play a mediating role in linking the response to mechanical treatment and cell fate reprogramming potential. Our results are consistent with recent studies, which reported that mechanical force from matrix stiffness can promote cell transformation to a transient state, accompanied by an increase in chromatin accessibility (Stowers et al., 2019; Walker et al., 2021). To the best of our knowledge, our study is the first report describing the modulation of mechanical properties to improve SCNT reprogramming efficiency. In the future, it will be worthwhile to combine the mechanical strain treatment with other known approaches to investigate whether multifactorial effects can further improve the SCNT reprogramming efficiency.

Recent studies indicated that the level of H3K9me3, a well-characterized heterochromatin mark, can be drastically decreased upon mechanical treatment (Le et al., 2016; Nava et al., 2020) and that erasing H3K9me3 can make chromatin more active in response to force (Sun et al., 2020). However, in our study, we observed that the level of H3K9me3 did not decrease globally upon mechanical strain treatment, suggesting the complexity of multifaceted chromatin responses to mechanical treatment. The diversity of chromatin responses upon mechanical treatments may be due to differences in the mechanical treatment type, strength, and duration used, together with cell type.

Our study clearly demonstrated the usefulness of cell state transition signature analysis (Zhu et al., 2017) in predicting cell state transition potential. Those signatures were derived from time-series gene expression data during cell state transitions, and at least some genes in each signature can reflect the features of intermediate states of a given transition process, which are usually transient but can be informative for revealing cell fate determination potential. Cell state transition signature analysis can be applied in future studies to facilitate the identification of ideal conditions for inducing cell fate transition.

## Experimental procedures

### Resource availability

#### Corresponding author

Further information and requests for resources and reagents should be addressed to the lead contact, Yong Zhang (yzhang@tongji.edu.cn).

#### Materials availability

No unique reagents were generated in this study.

## Author contributions

Y.Z. conceived the project; Y.C. and Y.Z. designed the research strategy; Y.C., C.L., R.X., S.Z., and Z.H. performed experiments under the supervision of S.G., Y.S., J.L., and X.L.; C.Z., Y.H., and Y.X. performed computational analysis; Y.C. and Y.Z. wrote the manuscript.

## Data Availability

All ATAC-seq, ChIP-seq, and RNA-seq datasets generated in this study are summarized in Table S1 and have been deposited in the Genome Sequence Archive (https://bigd.big.ac.cn/gsa/) under GSA: CRA005906. The details of experimental procedures are provided in the supplemental information.

## Supplemental information

Document S1. Supplemental experimental procedures, Figures S1–S4, and Tables S2–S4

Table S1. List of all performed datasets including RNA-seq, ATAC-seq, and ChIP-seq

Document S2. Article plus supplemental information
