# Supplementary material for: Mechanical strain treatment improves nuclear transfer reprogramming efficiency by enhancing chromatin accessibility
Source: Stem Cell Reports. 2023 Mar 23;18(4):807–16. doi: 10.1016/j.stemcr.2023.02.007 (PMC10147550; doi:10.1016/j.stemcr.2023.02.007)
Supplement: Document S1. Supplemental experimental procedures, Figures S1–S4, and Tables S2–S4 [file mmc1.pdf]

Stem Cell Reports, Volume *18*

Supplemental Information

Mechanical strain treatment improves nuclear transfer reprogramming efficiency by enhancing chromatin accessibility

Yujie Chen, Ruimin Xu, Shuang Zhou, Chengchen Zhao, Ziyue Hu, Yuwei Hua, Yanhong Xiong, Xiaoyu Liu, Junhong Lü, Yao Sun, Chong Li, Shaorong Gao, and Yong Zhang

SUPPLEMENTARY FIGURE

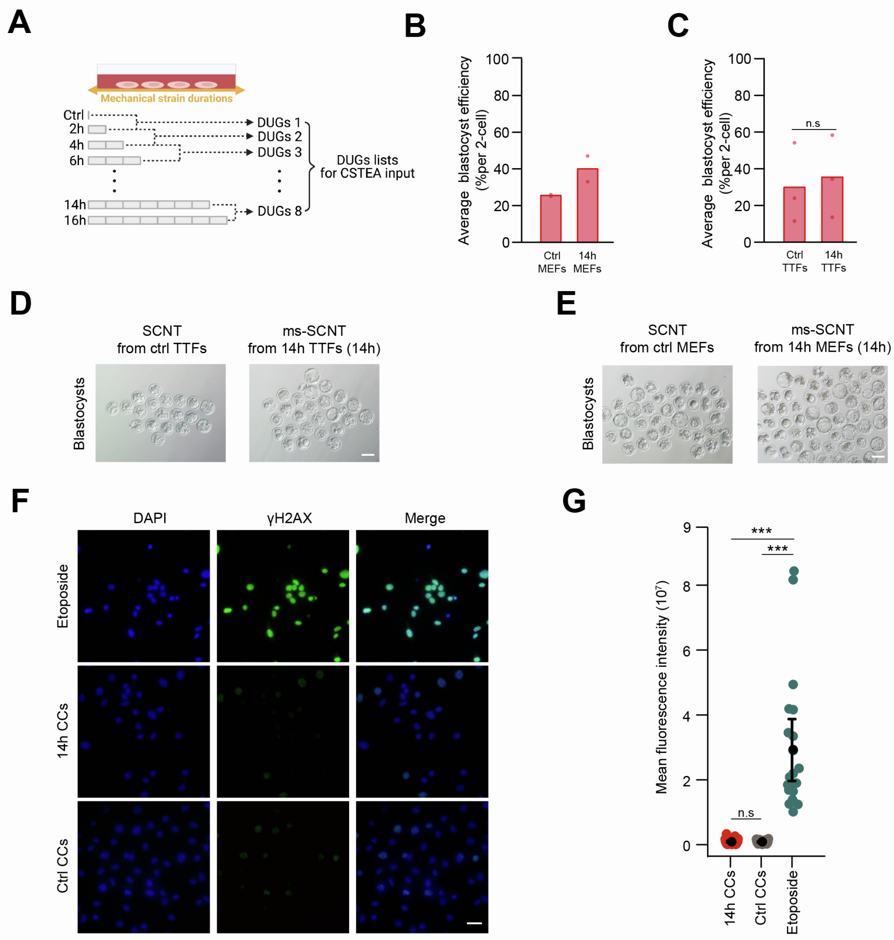

Supplementary Figure 1. Mechanical strain treatment improved the efficiency of SCNT using CCs and other cell types. (A) The workflow for identifying differentially upregulated genes (DUGs) for signature querying in CSTEA (n≥2), and this figure was created with BioRender (https://app.biorender.com). (B, C) The blastocyst developmental efficiency of 14 h mechanical strain-treated mouse embryonic fibroblast cell (MEF) (n=2) (B) and tail-tip fibroblast cell (TTF) (n=3) -derived ms-SCNT embryos (C). (D, E) The blastocyst phenotype of MEFs (D) and TTFs (e) 14 h ms-SCNT blastocyst phenotype. Scale bar, 100 μm. (F) Representative images of γH2AX staining in control and 14 h mechanical strain-treated CCs, with Etoposide-treated sample as the positive control. Scale bar, 50 μm (n=3). (G) Quantification of signals in γH2AX staining. Significant differences, ***$p < 0.001$.

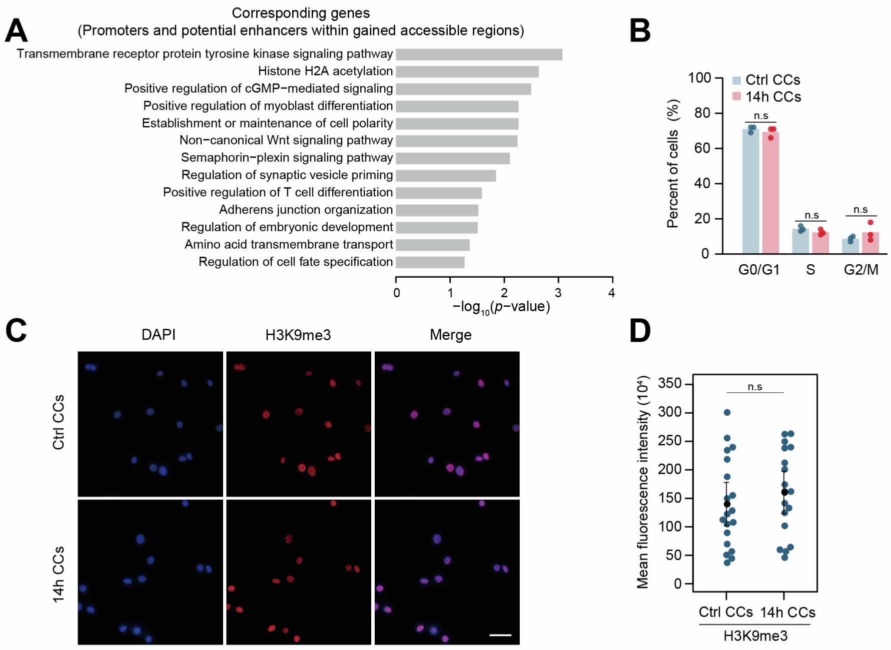

Supplementary Figure 2. Mechanical strain treatment did not globally decrease the H3K9me3 signal in CCs. (A) GO enrichment analysis of the genes whose promoters and potential enhancers overlapped with gained accessibility regions. The *p* value was calculated with Fisher's exact test. (B) The bar plot demonstrates the cell cycle distribution in control and 14 h mechanical strain-treated CCs (n=3). (C) Representative images of H3K9me3 staining in control and 14 h mechanical strain-treated CCs (n=3). Scale bar, 50 μm. (D) The quantification of positive signals in H3K9me3 staining.

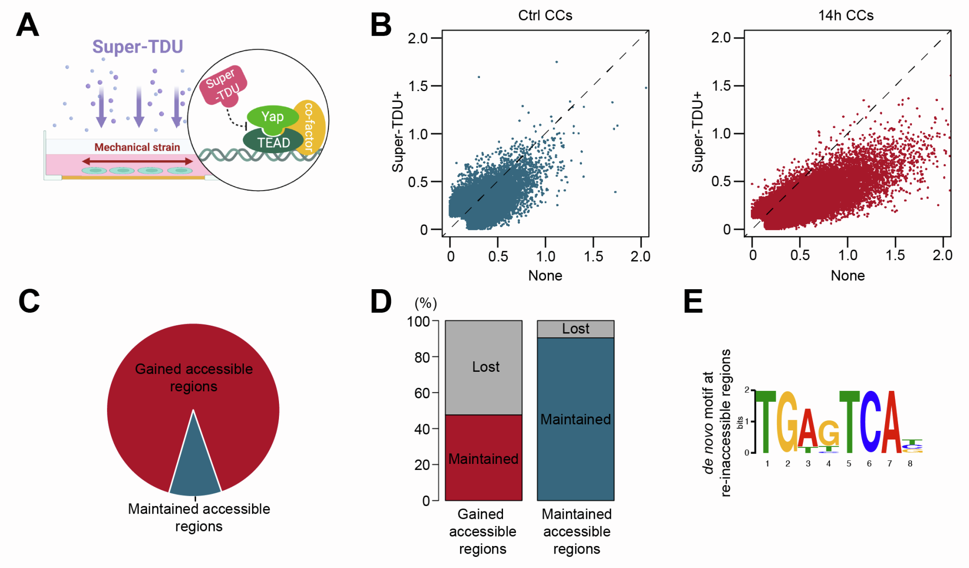

Supplementary Figure 3. YAP contributed to mechanical strain-induced increases in chromatin accessibility. (A) Schematic of the super-TDU function, and this figure was created with BioRender (https://app.biorender.com). (B) Scatterplots of normalized ATAC-seq signals of control CCs with and without Super-TDU (left) and mechanical strain-treated CCs with and without Super-TDU (right) (n=2). (C) Pie chart showing the proportion of original categories for the regions losing accessibility upon super-TDU treatment. (D) The stacked bar plot shows the proportion of regions with maintained and lost accessibility upon super-TDU treatment. (E) The sequence logo of the *de novo* discovered motif in re-inaccessible regions.

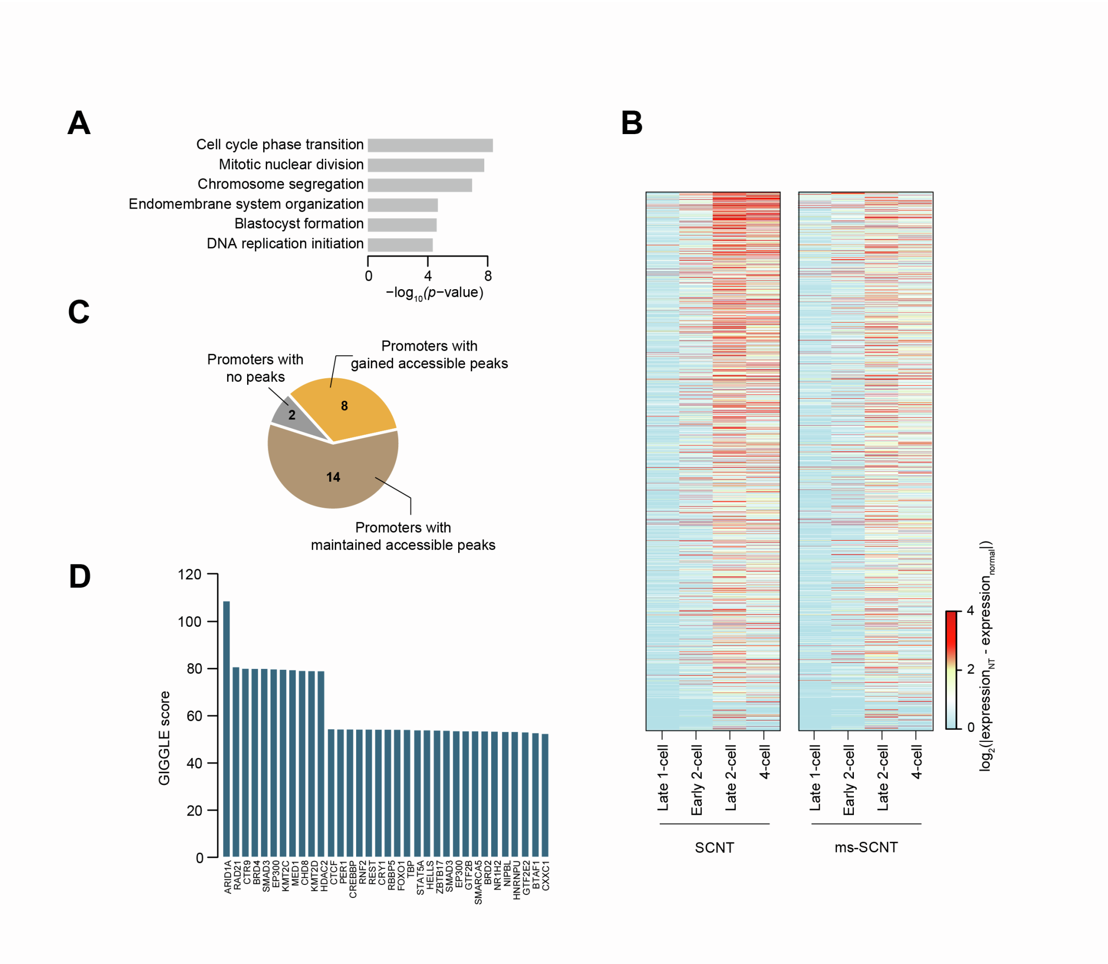

Supplementary Figure 4. Regions with mechanical strain-induced gains in accessibility affected ms-SCNT embryo development. (A) GO enrichment analysis of genes with repaired downregulation. The *p* value was calculated by Fisher's exact test. (B) The heatmap demonstrates the expression level differences of nonmaternally loaded EGA genes between normal *in vivo* fertilized embryos and ms-SCNT embryos (n≥2). (C) Pie chart demonstrating the proportion of 24 nonmaternally loaded EGA genes with gained or maintained promoter accessibility in mechanical strain-treated CCs. (D) Bar plot demonstrating the chromatin regulators predicted by the Cistrome DB Toolkit.

Table S1: List of all performed datasets including RNA-seq, ATAC-seq and ChIP-seq

This table is provided as a separate Excel sheet.

Table S2: The E-values of AP-1-related factor motifs in re-inaccessible regions, related to Figure3.

Factors	E-value
FOSB	3.75×10^{-7}
ATF3	3.40×10^{-6}
FOSL2	4.48×10^{-5}
JUN	6.24×10^{-5}

Table S3: Table of the overlap between downregulated genes in SCNT and ms-SCNT embryos, related to Figure 4

Stages	Down regulated genes			
	SCNT	ms-SCNT	Overlapped	*p*-value
Late 1-cell	1152	375	243	2.92×10^{-232}
Early 2-cell	1112	497	300	3.43×10^{-281}
Late 2-cell	722	268	123	4.52×10^{-116}
4-cell	606	297	141	5.47×10^{-148}

Table S4: Table showing the statistical significance of EGA genes' expression differences between SCNT / ms-SCNT and normal embryos. The *p* value was calculated using the paired Wilcoxon test, related to Figure 4

	Late 1-cell	Early 2-cell	Late 2-cell	4-cell
SCNT vs. normal	1.11×10^{-4}	2.44×10^{-107}	1.45×10^{-232}	2.16×10^{-50}
ms-SCNT vs. normal	7.74×10^{-4}	6.62×10^{-16}	1.08×10^{-72}	1.45×10^{-51}

SUPPLEMENTAL EXPERIMENTAL PROCEDURE

Mice

BDF1 (C57BL/6 × DBA/2) and ICR mice were housed in the specific pathogen-free-grade animal facility of Tongji University. All mouse maintenance and experiments were performed following the Tongji University Laboratory Animals Procedures. Cells were isolated from our mouse strain. Briefly, cumulus cells (CCs) and tail-tip fibroblast cells (TTFs) were isolated from BDF1 mice, and mouse embryonic fibroblast cells (MEFs) were isolated and cultured from ICR mouse embryos at 13.5 dpc.

Cell culture and mechanical strain treatment

Approximately 1.2 × 10^5 cells per well were seeded on a 6-well BioFlex® culture plate with a silicon elastomer membrane, and the cells were cultured in medium (Sigma) supplemented with glutamine (Millipore) and penicillin–streptomycin (Gibco). Cells were seeded on plates for at least 2 h to allow cells to attach stably before being stretched, and changing the medium before stretching was performed as recommended. Then, the cells were stretched under cyclic mechanical strain using the Flexcell Tension System (FX5000T; Flexcell International Corporation) at 7.5% and a frequency of 0.5 Hz for all durations. To interfere with the YAP-TEAD interaction, cells were exposed to 9.5 μmol Super-TDU (Selleck) before stretching began.

SCNT and embryo culture

SCNT was carried out according to the procedures described in our previous work(Gao et al., 2018). Briefly, 8~10-week-old female BDF1 mice were injected with 5 units of pregnant mare serum gonadotropin (PMSG), followed after 48 h by 6 units of human chorionic gonadotropin (hCG, San-Sheng Pharmaceutical). MII oocytes were isolated from the dissected oviducts 13 h later and transferred into prewarmed Chatot–Ziomek–Bavister medium (CZB). CCs were collected into 1.5 mL tubes from the oocyte-cumulus complex after hyaluronidase (Sigma) treatment at 37 °C in 5% CO_2, followed by two washes with HEPES-CZB. All MII oocytes were enucleated in HEPES-CZB containing 5 μg/mL cytochalasin B (Sigma) and kept at 37 °C and 5% CO_2 until SCNT. The nuclei of mechanical strain-treated and control donor cells were injected into enucleated MII oocytes with a piezo-driven micromanipulator (Eppendorf). The reconstructed embryos were cultured in CZB for 1 h and activated in calcium-free CZB containing 1% $SrCl_2$ for 5 h. All reconstructed embryos were transferred to G-1 PLUS medium (Vitrolife) for further culture during preimplantation development.

Immunofluorescence staining in embryos and cells

For preimplantation embryo immunofluorescence staining, blastocysts were fixed in 4% paraformaldehyde for 1 h at room temperature and washed twice for 15 min with 0.5% BSA-PBS. All fixed embryos were incubated in anti-OCT4 (Santa Cruz Biotechnology) and anti-CDX2 (Abcam) antibody at 4 °C overnight in 0.5% BSA-PBS containing 0.1% Triton and washed twice for 15 min with 0.5% BSA-PBS at room temperature. The incubation with conjugated secondary antibody (Invitrogen) was performed in 0.5% BSA-PBS at room temperature for 1 h; then, the cells were washed twice, incubated with DAPI for 15 min at room temperature, and imaged. For cell immunofluorescence staining, cells were incubated in anti-

H3K9me3 (Active Motif) or γH2AX (Beyotime) antibody, and the positive control in γH2AX immunofluorescence staining was treated with the medium containing 100 μM etoposide (Selleck) for 1.5 h. All washing steps and DAPI incubation were performed for 5 min, and the other procedures were as described above.

DNaseI-TUNEL

Before DNase I treatment, cells seeded in BioFlex® culture plates were washed twice with 1X calcium-free PBS and incubated with PBS containing 0.2% Triton X-100 for 5 min followed by two washes. Cells were incubated with 200 μL of fresh DNase I buffer (40 mM pH 7.9 Tris-HCl, 10 mM NaCl, 6 mM $MgCl_2$, 10 mM $CaCl_2$) for 5 min and then replaced with buffer containing 5.5 units/mL DNase I (Roche) for 10 min of incubation at room temperature. After DNase I treatment, the cells were fixed immediately with 1× paraformaldehyde buffer (Sangon Biotech) for 10 min and washed twice with PBS. The cells were preincubated at room temperature for 5~10 min with 200 μL equilibration buffer (25 mM pH 7.9 Tris-HCl, 20 mM potassium cacodylate, 0.2 mM DTT, and 0.25 mg/mL BSA), and then the TDT reaction buffer was replaced with 7 μL nucleotide mix, 1.5 μL rTDT (Promega) and 63 μL equilibration buffer at 37 °C for 1 h. The bottom membranes around the edges of the wells were cut out and then washed for 5 min by immersion in PBS. The membranes were then immersed in 20 mL 2× SSC (Promega) for 15 min, washed 3 times with PBS, incubated with DAPI for 5 min and imaged.

ATAC-seq

To prepare the nuclei, 1 × 10^5 cells were harvested per BioFlex® culture plate well, and the cells were spun down at 500 × g for 5 min, followed by washing with 200 μL of cold 1× PBS. Cells were lysed in 50 μL of fresh and cold lysis buffer (10 mM pH 7.4 Tris-HCl, 10 mM NaCl, 3 mM $MgCl_2$, 0.1% IGEPAL CA-630), gently pipetted several times, and then placed on ice for 10 min. The samples were immediately spun in a refrigerated centrifuge at 500 × g for 5 min, the supernatants were carefully removed, and the precipitates were kept on ice. For the following fragmentation step, the precipitates were resuspended in 20 μL fragment master mix (4 μL 5× TTBL, 5 μL TTE mix (Vazyme), 11 μL nuclease-free water) and gently pipetted several times on ice; then, the reaction was carried out at 37 °C for 30 min. The reaction was directly stopped by adding 5 μL of 5× TS (Vazyme) and transferring the reaction to room temperature for 5 min. Then, and the samples were purified with AMPure XP beads. To generate the amplified library, the primers and the master mix (Vazyme) were mixed with the fragments, amplified for a total of 8-10 cycles, and purified with 1.2× AMPure XP beads.

Native ChIP-seq

A total of 1 × 10^5 cells were washed twice with 1× PBS and resuspended in 20 μL cold nuclear extraction buffer (10 mM pH 8.5 Tris-HCl, 140 mM NaCl, 5 mM $MgCl_2$, 0.6% NP-40, 0.1 mM phenylmethylsulfonyl fluoride, and 1 × protease inhibitor cocktail (PIC)). thoroughly mixed, and incubated on ice for 1 min. The cells were then spun in a refrigerated centrifuge at 4000 rpm for 5 min, and carefully absorbed supernatants away. MNase (NEB) was diluted to 10 U/μL with MNase dilution buffer (10 mM pH 7.5 Tris-HCl, 50 mM NaCl, 1 mM ethylenediaminetetraacetic acid, 50% glycerin), and MNase master buffer was prepared (10 mM pH 7.5 Tris-HCl, 1 mM $CaCl_2$); then, 40 μL of MNase master mix (5 μL MNase master buffer, 2 mM dithiothreitol, 5%

PEG6000, 3 μL 10 U/μL MNase) was used to fully resuspend the precipitates, followed by a 5 min incubation at 37 °C. A 5.5 μL aliquot of MNase stop buffer (1:4 0.5 mM ethylenediaminetetraacetic acid:nuclease-free water) was used to stop the reaction, and 5.5 μL nuclear break buffer (1% deoxycholate and 1% Triton X-100) was added to release the chromatin. Chromatin was pre-cleared with 10 μL of 1:1 protein A:protein G Dynabeads (Life Technologies) at 4 °C for 1 h. One milligram of H3K9me3 antibody (Active motif no. 39161) was pre-incubated with Dynabeads, and the antibody-beads complex was mixed with the chromatin sample and incubated overnight at 4 °C. The samples were washed twice with 200 μL low-salt washing buffer (20 mM pH 8.0 Tris-HCl, 0.1% SDS, 1% Triton X-100, 2 mM ethylenediaminetetraacetic acid, 150 mM NaCl, 1 ×PIC), and twice with 200 μL high-salt washing buffer (20 mM pH 8.0 Tris-HCl, 0.1% SDS, 1% Triton X-100, 2 mM ethylenediaminetetraacetic acid, 500 mM NaCl, 1 ×PIC). IP material was eluted from the beads with 100 μL hot elution buffer (100 mM $NaHCO_3$ and 1% SDS) at 65 °C for 1.5~2 h, and the supernatants were transferred to a new tube. Then, the IP material was treated with 2.5 μL 20 mg/mL proteinase K at 55 °C for 30 min, purified with phenol chloroform, ethanol-precipitated and resuspended in 10 mM pH 8.0 Tris-HCl. DNA libraries were constructed with KAPA Hyper Prep Kit. In brief, samples were end-repaired with A-tailing, adapter ligated and amplified for 10 cycles. Libraries were purified with 1.2× AMPure XP beads.

RNA-seq

For the RNA-seq performed in CCs, in brief, cells were lysed, followed immediately by the reverse transcription process to generate first-strand cDNAs, 3' poly(A) tailing, and adapter ligation. The adapter-ligated cDNAs were amplified for 18 cycles for cDNA library construction with KAPA Hyper Prep Kit, followed by AMPure XP bead purification. For the single-embryo RNA-seq, late 1-cell, early 2-cell, late 2-cell and 4-cell reconstructed embryos were harvested. cDNA libraries were synthesized and amplified using SMARTer Ultra Low Input RNA Kit for sequencing following the manufacturer's instructions. Fragments of 0.5-5 kb were selected by 2% agarose gel, and cDNA purification was performed using a QIAquick PCR Purification Kit.

Sequencing data processing

ATAC-seq reads were mapped to the mm10 reference genome after trimming the adaptors using bowtie2 (v2.3.5.1, the parameters used were --trim-to 3:40 -x mm10)(Langmead and Salzberg, 2012), and SAMtools(Li et al., 2009) was used to remove the repetitive, lower quality (Q≤30) and mitochondrial DNA reads from the total mapped reads. Reads with lengths less than 50 base pairs (bp) were retained for subsequent analysis. To allow the comparison of data with different sequencing depths, the signals were normalized to ten million reads for each sample, and the values were further compressed into a binary format (bigWig) for downstream analysis and data visualization. Peak calling was performed using MACS (v1.4.2)(Zhang et al., 2008) with the following parameters: -g mm --keep-dup all --nomodel --shiftsize 25.

H3K9me3 ChIP-seq read mapping was carried out as previously described, and the peaks were called using MACS (v2.1.3)(Zhang et al., 2008) with parameters as follows: -g mm -p 1e-5 --broad --broad-cutoff 1e-5 --keep-dup all. The signals were normalized to twenty million reads for each sample. The CC and embryo RNA-seq reads were mapped to the mm10 reference

genome with HISAT2 (v2.1.0)(Kim et al., 2019), and StringTie (v1.3.3b)(Pertea et al., 2015) was used to quantify the transcription level of each gene in each sample as FPKM (fragments per kilobase of exon per million mapped fragments).

Prediction of mechanically induced cell fate transition potential

Given the FPKM matrix generated above, GFOLD(Feng et al., 2012) was used to perform differential expression analysis between adjacent duration-conditions, and the genes with a GFOLD value > 0.5 were considered differentially upregulated genes (DUGs). The details are provided in the Supplemental Information. The numbers of all DUGs from every paired treatment condition were counted, and the DUGs that appeared at least twice in all adjacent conditions were considered as features for subsequent analysis. Default parameters in CSTEA(Zhu et al., 2017) were used for signature querying of these DUGs, and the top five queried processes are shown. The brief description of cell state transition signature analysis of CSTEA is as follows. For each publicly available time-series gene expression data, CSTEA defined its cell state transition signature as the union of differentially expressed genes at two consecutive time points. If two or more datasets with the same origin and destination cell types were available, their signatures were combined. Given a querying gene set, its enrichment analysis was performed using Fisher's exact test against all defined cell state transition signatures. The link of CSTEA webserver is https://mai.fudan.edu.cn/cstea/.

Genome annotation

Promoters were defined as the regions ± 2 kb around the transcription start sites (TSSs) of RefSeq genes, and potential enhancers were defined as the union set of public H3K27ac ChIP-seq peaks and H3K4me1 ChIP-seq peaks in mice early embryos, excluding the promoters. We downloaded the public ChIP-seq data from Gene Express Omnibus (GEO). H3K27ac ChIP-seq data included GSE185653 (zygote, early 2-cell, late 2-cell, 4-cell), GSE207222 (zygote, early 2-cell, late 2-cell), GSE125318 (E6.5) and GSE195592 (E8.4). H3K4me1 ChIP-seq data included GSE98101 (E6.5, E7.0, E7.5), GSE94131 (E8.5, E9.5) and GSE79941 (E9.5). Peak calling was performed using MACS (v1.4.2)(Zhang et al., 2008) with the default parameters. We merged the overlapping peaks, and defined these peaks as potential enhancers in mice. If a potential enhancer is located within ± 10 kb of a gene's TSS, it was defined as the gene's potential enhancer. If a gained accessible chromatin region was located within the promoter or potential enhancer of a gene, that gene was regarded as corresponding to the gained accessible chromatin region.

Functional annotation analysis

Functional annotation was performed using the Database for Annotation, Visualization and Integrated Discovery (DAVID) Bioinformatics Resource 6.8(Huang et al., 2009). Gene Ontology terms for each functional cluster were summarized to a representative term, and *p* values were plotted to show the significance.

Nonmaternally loaded EGA genes

The list of 1,946 nonmaternally loaded EGA genes was defined as follows: high transcription level (FPKM > 5) in 2-cell stage embryos and low transcription level (FPKM < 1) in MII oocytes. Public data (GSE71434) were used for this analysis.

Motif analysis

Motif analysis was performed using MEME suite tools (v5.0.5)(Bailey et al., 2015) against the genome sequence of the given ATAC-seq peak regions (peak summits ± 100 bp) with the following parameters: -maxsize 20000000 -mod zoops -nmotifs 30 -minw 6 -maxw 50 -revcomp. Then, TOMTOM within the MEME suite was applied to test the similarity between the identified motifs and the binding motifs of known factors with the following parameters: -verbosity 1 -min-overlap 5 -dist pearson -evalue -thresh 10.

Chromatin regulator binding prediction

ChIP-seq peaks of all mouse chromatin regulators were downloaded from the Cistrome Data Browser(Zheng et al., 2019), and the transcription factors were displayed when they overlapped with the promoters (TSS ± 2k) of the 24 genes shown in Figure 4D. The GIGGLE score was calculated by the Cistrome DB Toolkit; chromatin regulators with values greater than 50 (the default confidence of the Cistrome DB Toolkit) were displayed, and redundancy was removed.

References

Bailey, T.L., Johnson, J., Grant, C.E., and Noble, W.S. (2015). The MEME Suite. Nucleic Acids Res 43, W39-49.

Gao, R., Wang, C., Gao, Y., Xiu, W., Chen, J., Kou, X., Zhao, Y., Liao, Y., Bai, D., Qiao, Z., et al. (2018). Inhibition of Aberrant DNA Re-methylation Improves Post-implantation Development of Somatic Cell Nuclear Transfer Embryos. Cell Stem Cell 23, 426-435 e425.

Huang, D.W., Sherman, B.T., and Lempicki, R.A. (2009). Systematic and integrative analysis of large gene lists using DAVID bioinformatics resources. Nat Protoc 4, 44-57.

Kim, D., Paggi, J.M., Park, C., Bennett, C., and Salzberg, S.L. (2019). Graph-based genome alignment and genotyping with HISAT2 and HISAT-genotype. Nat Biotechnol 37, 907-915.

Langmead, B., and Salzberg, S.L. (2012). Fast gapped-read alignment with Bowtie 2. Nat Methods 9, 357-359.

Li, H., Handsaker, B., Wysoker, A., Fennell, T., Ruan, J., Homer, N., Marth, G., Abecasis, G., Durbin, R., and Genome Project Data Processing, S. (2009). The Sequence Alignment/Map format and SAMtools. Bioinformatics 25, 2078-2079.

Pertea, M., Pertea, G.M., Antonescu, C.M., Chang, T.C., Mendell, J.T., and Salzberg, S.L. (2015). StringTie enables improved reconstruction of a transcriptome from RNA-seq reads. Nat Biotechnol 33, 290-295.

Zhang, Y., Liu, T., Meyer, C.A., Eeckhoute, J., Johnson, D.S., Bernstein, B.E., Nusbaum, C., Myers, R.M., Brown, M., Li, W., et al. (2008). Model-based analysis of ChIP-Seq (MACS). Genome Biol 9, R137.

Zheng, R., Wan, C., Mei, S., Qin, Q., Wu, Q., Sun, H., Chen, C.H., Brown, M., Zhang, X., Meyer, C.A., et al. (2019). Cistrome Data Browser: expanded datasets and new tools for gene regulatory analysis. Nucleic Acids Res 47, D729-D735.
