# Supplementary material for: Mechanical strain treatment improves nuclear transfer reprogramming efficiency by enhancing chromatin accessibility
Source: Stem Cell Reports. 2023 Mar 23;18(4):807–16. doi: 10.1016/j.stemcr.2023.02.007 (PMC10147550; doi:10.1016/j.stemcr.2023.02.007)
Supplement: Document S2. Article plus supplemental information [file mmc3.pdf]

Stem Cell Reports
Report

OPEN ACCESS

Mechanical strain treatment improves nuclear transfer reprogramming efficiency by enhancing chromatin accessibility

Yujie Chen,[1,6] Ruimin Xu,[1,6] Shuang Zhou,[2,6,7] Chengchen Zhao,[1,6,8] Ziyue Hu,[2] Yuwei Hua,[1] Yanhong Xiong,[1] Xiaoyu Liu,[1] Junhong Lü,[3,4] Yao Sun,[2,*] Chong Li,[5,*] Shaorong Gao,[1,5,*] and Yong Zhang[1,*]

[1]Institute for Regenerative Medicine, Shanghai East Hospital, Shanghai Key Laboratory of Signaling and Disease Research, Frontier Science Center for Stem Cell Research, School of Life Sciences and Technology, Tongji University, Shanghai 200092, China

[2]Department of Implantology, School & Hospital of Stomatology, Tongji University, Shanghai, China

[3]Shanghai Advanced Research Institute, Chinese Academy of Sciences, Shanghai 201203, China

[4]College of Pharmacy, Binzhou Medical University, Yantai 264003, China

[5]Shanghai Key Laboratory of Maternal Fetal Medicine, Shanghai Institute of Maternal-Fetal Medicine and Gynecologic Oncology, Clinical and Translation Research Center, Shanghai First Maternity and Infant Hospital, Frontier Science Center for Stem Cell Research, School of Life Science and Technology, Tongji University, Shanghai 200092, China

[6]These authors contributed equally

[7]Present address: Department of Stomatology, Children's Hospital of Soochow University, Suzhou, Jiangsu 215025, China

[8]Present address: School of Life Sciences, Westlake University, Hangzhou, Zhejiang 310012, China

*Correspondence: yaosun@tongji.edu.cn (Y.S.), lichong@tongji.edu.cn (C.L.), gaoshaorong@tongji.edu.cn (S.G.), yzhang@tongji.edu.cn (Y.Z.)

https://doi.org/10.1016/j.stemcr.2023.02.007

SUMMARY

Cellular mechanical properties are considered to be important factors affecting cell fate transitions, but the links between cellular mechanical properties and transition efficiency and chromatin structure remain elusive. Here, we predicted that mechanical strain treatment could induce signatures of cellular dedifferentiation and transdifferentiation, and we validated this prediction by showing that mechanical strain-treated mouse cumulus cells (CCs) exhibit significantly improved somatic cell nuclear transfer (SCNT) reprogramming efficiency. We found that the chromatin accessibility of CCs was globally increased by mechanical strain treatment and that this increase was partially mediated by the induction of the YAP-TEAD interaction. Moreover, using mechanical strain-treated CCs could prevent transcriptional dysregulation in SCNT embryos. Taken together, our study results demonstrated that modulating cell mechanical properties to regulate epigenetic status is a promising approach to facilitate cell fate transition.

INTRODUCTION

Recently, cellular mechanical properties have been demonstrated to play important roles in multiple cell fate determination events, including stem cell lineage commitment (Engler et al., 2006; McBeath et al., 2004), differentiation or self-renewal induction (Chowdhury et al., 2010; Connelly et al., 2010; Gilbert et al., 2010), and cell allocation patterns during development (Chan et al., 2019). Although extracellular forces could be transmitted to the nucleus interior and lead to chromatin stretching and the expression of reporter genes (Tajik et al., 2016), applying cellular mechanical properties to induce cell fate transitions is still challenging, mainly because identifying the appropriate extracellular force to effectively facilitate cell fate transition is a time-consuming and labor-intensive process.

We aimed to overcome this challenge by considering the following two issues. First, we relied on previously collected time-series gene expression data during cell state transitions and defined gene signatures for those processes (Zhu et al., 2017). These gene signatures of cell state transitions could be used to evaluate the directed reprogramming potential of cells upon extracellular force treatments by comparing their differentially expressed genes, and this evaluation procedure can accelerate the fine-tuning of the effective extracellular force. Second, somatic cell nuclear transfer (SCNT) is an ideal system in which to validate the extracellular force-induced reprogramming for mouse cells, as the measurement of the effectiveness is quick and clear, i.e., the rate of blastocyst formation at day 3.5 post nuclear transfer. It has been reported that SCNT reprogramming efficiency can be improved by altering the epigenetic status of donor nuclei (Matoba and Zhang, 2018). However, to the best of our knowledge, there has been no report on the use of mechanical property modulation to improve SCNT reprogramming efficiency. In this study, we applied an integrative approach to predict that mechanical strain treatment could induce signatures of cellular dedifferentiation and transdifferentiation and validated this prediction by showing that mechanical strain-treated mouse somatic cells exhibit significantly improved SCNT reprogramming efficiency, which demonstrated that modulating mechanical properties to regulate epigenetic status is a promising approach to achieve cell fate transition.

This is an open access article under the CC BY license (http://creativecommons.org/licenses/by/4.0/).

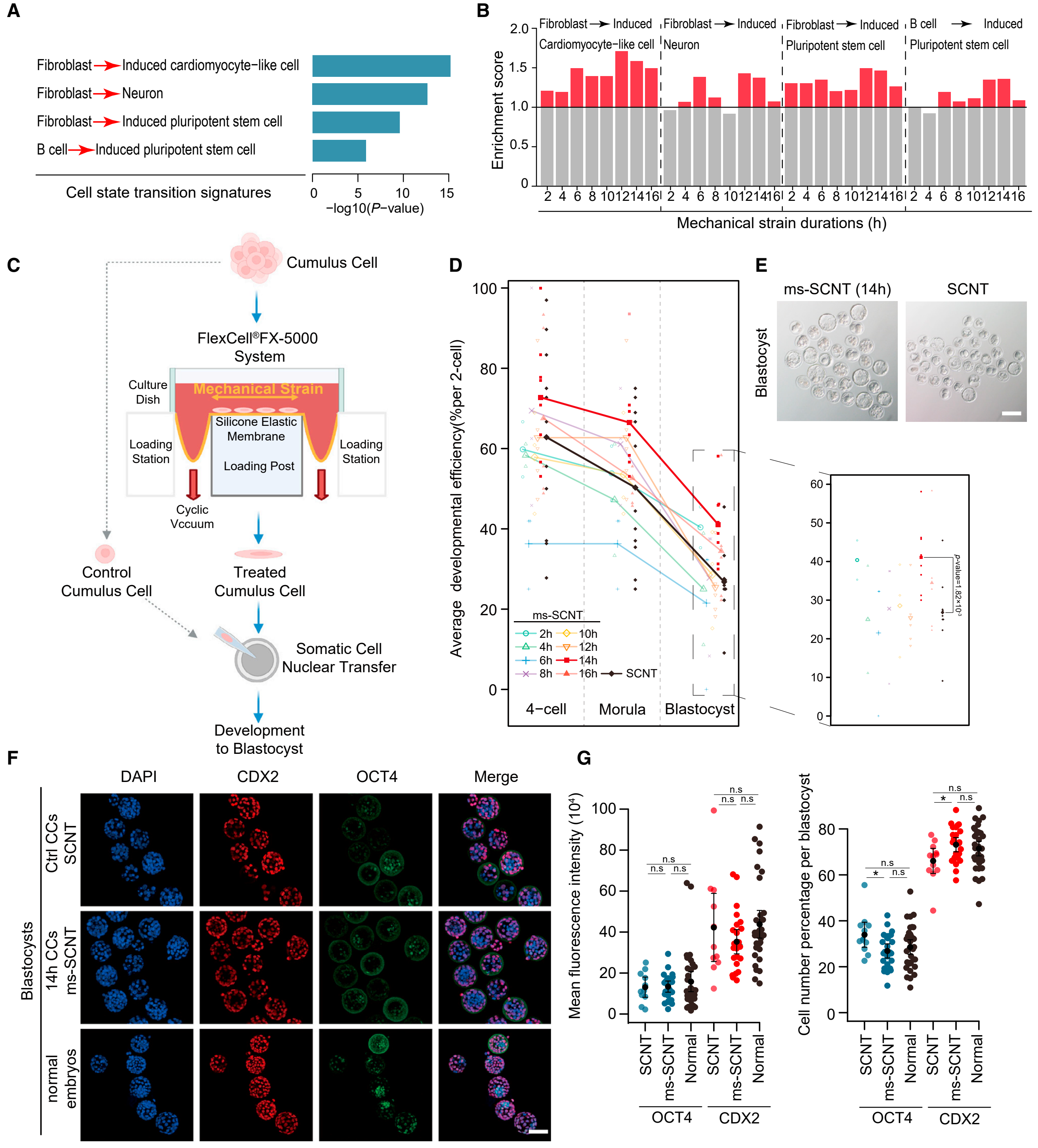

Figure 1. Mechanical strain treatment improved SCNT efficiency

(A) The bar plot demonstrates the top four cell state transition signatures identified by CSTEA.

(B) The bar plot demonstrates the signature enrichment in each duration, and those with an enrichment score <1 (gray) were considered not enriched.

(C) The ms-SCNT embryo procedure, created with BioRender (https://app.biorender.com).

(D) The developmental efficiency of ms-SCNT embryos across different mechanical strain durations and SCNT embryos (n $\geq$ 2).

(legend continued on next page)

RESULTS

Mechanical strain treatment improved SCNT efficiency

To investigate whether mechanical strain treatment can facilitate cell fate transition, we exposed mouse cumulus cells (CCs), which are common SCNT donors, to a biaxial cyclic mechanical strain of 7.5% at 0.5 Hz for durations ranging from 2 to 16 h, with 2 h intervals, and performed RNA sequencing (RNA-seq) for each condition (Figure S1A; Table S1). Since over-expressing reprogramming factors can facilitate cell fate transitions, and since activators may play central roles in reconstructing transcriptional networks (Graf and Enver, 2009), we focused on differentially upregulated genes to assess the cellular reprogramming potentials. Differentially upregulated genes between samples of adjacent durations were grouped, and the cell state transition signature analysis (Zhu et al., 2017) revealed that these genes were highly enriched in the signatures of cellular dedifferentiation and transdifferentiation (Figure 1A; see experimental procedures for details). For each mechanical strain duration, we identified differentially upregulated genes between mechanical strain-treated and control CCs (gene numbers: 760 for 2 h; 857 for 4 h; 651 for 6 h; 970 for 8 h; 661 for 10 h; 521 for 12 h; 541 for 14 h; 607 for 16 h), which we regarded as mechanical strain-induced genes, and calculated their enrichment scores to identify highly enriched cell state transition signatures (see experimental procedures for details). The 12 and 14 h mechanical strain durations showed the highest enrichment scores in all signatures of cellular dedifferentiation and transdifferentiation (Figure 1B), suggesting that appropriate mechanical strain treatment might enhance the cellular reprogramming potential of CCs.

To confirm the enhancement of cellular reprogramming or transdifferentiation potential of mechanical strain-treated CCs, we performed SCNT using CCs exposed to mechanical strain for different durations as donors and obtained mechanical strain-treated SCNT (ms-SCNT) embryos (Figure 1C). The ms-SCNT embryos from several duration groups exhibited significantly higher rates of blastocyst formation than control SCNT embryos (26.8%), with the 14 h duration showing the highest rate (41.6%) (Figures 1D and 1E), consistent with the 14 h duration displaying one of the highest enrichment scores in terms of cellular dedifferentiation and transdifferentiation signatures. In addition to CCs, ms-SCNT embryos generated from 14 h mechanical strain-treated mouse embryonic fibroblast cells and tail-tip fibroblast cells displayed higher (statistically not significant) blastocyst formation rates (embryonic fibroblast cells: 25.40% for control, 40.14% for 14 h mechanical strain treatment; tail-tip fibroblast cells: 28.68% for control, 34.35% for 14 h mechanical strain treatment; Figures S1B–S1E), confirming the effectiveness of mechanical strain treatment for improving SCNT efficiency for other cell types. We next focused on ms-SCNT embryos based on CCs treated with mechanical strain for 14 h. We labeled inner cell mass (ICM) and trophectoderm (TE) cells with OCT4 and CDX2, respectively, and the immunofluorescence intensities of both markers showed no significant differences between ms-SCNT and normal blastocysts (Figures 1F and 1G), confirming the quality of the ms-SCNT blastocysts. To evaluate whether mechanical strain treatment can cause DNA damage, we performed γH2AX immunofluorescence staining for 14 h mechanical strain-treated, etoposide-treated, and control CCs, and no statistical differences were observed between mechanical strain-treated and control CCs (Figures S1F and S1G), suggesting that 14 h mechanical strain treatment may not cause DNA damage. Our results demonstrated the effectiveness of mechanical strain treatment on improving SCNT efficiency, at least up to the blastocyst stage.

Mechanical strain treatment increased chromatin accessibility in CCs

We next investigated the effects of 14 h mechanical strain treatment on CCs. As chromatin accessibility is closely related to gene transcription regulation and is sensitive to extracellular mechanical environments (Stowers et al., 2019), we suspected that mechanical strain treatment might alter the chromatin accessibility of the CCs. To test this assumption, we performed assay for transposase-accessible chromatin (ATAC)-seq in mechanical strain-treated and control CCs (Table S1). We observed a global increase in chromatin accessibility in mechanical strain-treated CCs (Figure 2A), with a large number of newly accessible chromatin regions gained upon mechanical strain treatment (38,228 and 92,419 accessible chromatin regions in control and mechanical strain-treated CCs, respectively). To confirm the global increase in chromatin accessibility upon mechanical strain treatment, we performed a DNase-TUNEL assay in mechanical strain-treated and control CCs. The fluorescence signals were much stronger in mechanical strain-treated CCs than in the control cells (Figures 2B and 2C), consistent with the ATAC-seq profiles. A total of 15.4% and 29.4% of gained accessible chromatin regions were located in promoters and potential enhancers

(E) The blastocyst phenotype of 14 h ms-SCNT and SCNT embryos. Scale bar, 100 μm.

(F) CDX2 and OCT4 staining in 14 h ms-SCNT, SCNT, and normal *in vitro* fertilization blastocysts (n = 3). Scale bar, 100 μm.

(G) Scatterplot demonstrating the quantification of positive CDX2 and OCT4 staining (left), and the percentage of positive cells in each blastocyst (right). Significant differences, *$p < 0.05$.

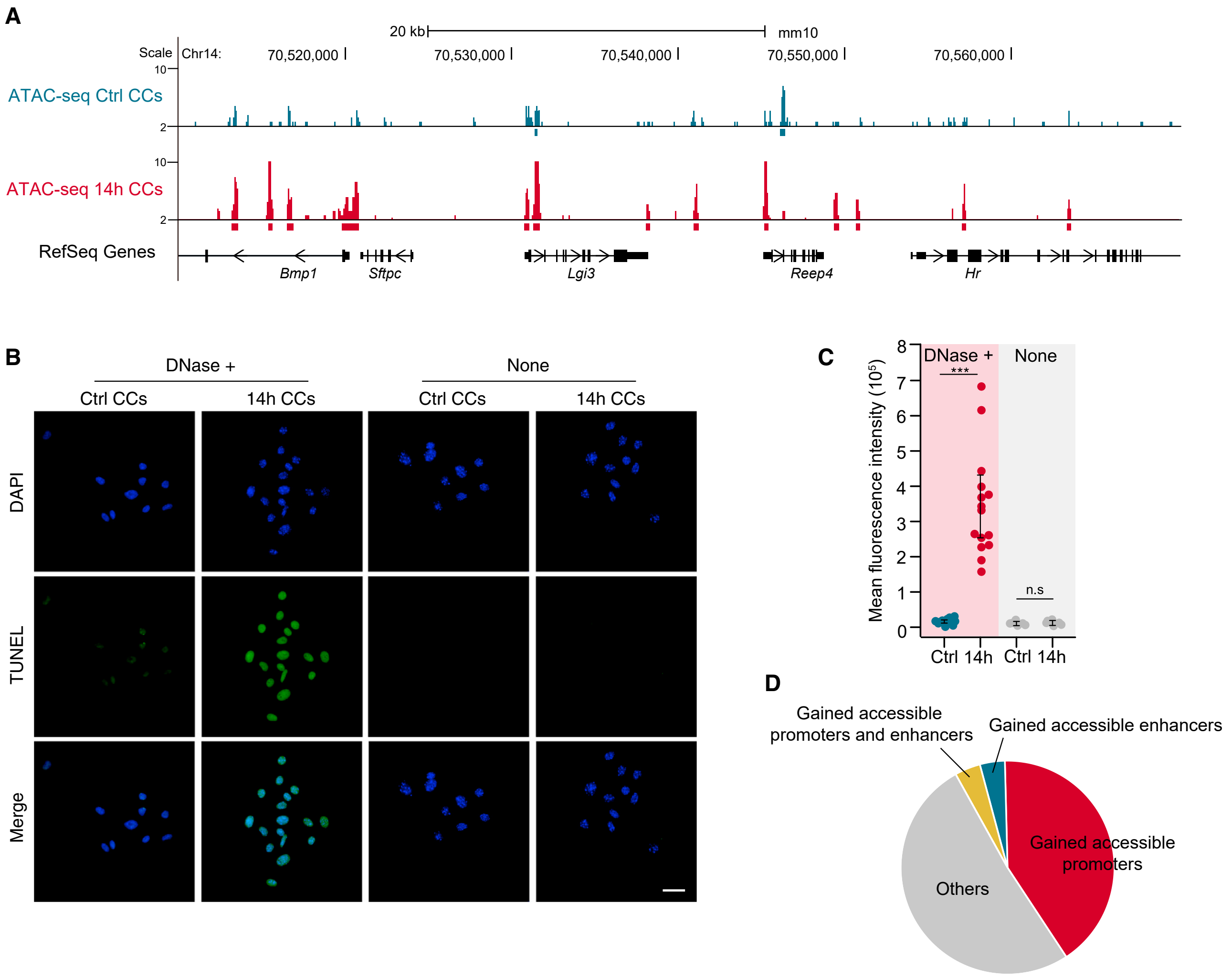

Figure 2. Mechanical strain treatment increased the chromatin accessibility of CCs

(A) Genome browser view of a representative region as an example to illustrate that the ATAC-seq signals increased at some promoter regions after 14 h of mechanical strain exposure (n = 2).

(B) TUNEL assay of the control and 14 h mechanical strain-treated CCs with or without DNase treatment (n = 3). Scale bar, 20 μm.

(C) Quantification of positive signals in the TUNEL assay with or without DNase treatment. ***p < 0.001.

(D) The pie chart demonstrates the percentage of upregulated genes associated with regions of chromatin accessibility gain in the 14 h mechanical strain-induced cells.

(see experimental procedures for details), 48.8% of mechanical strain-induced genes gained accessible promoters or potential enhancers, and those genes were functionally enriched in differentiation and developmental processes (Figures 2D and S2A). Our results demonstrated that mechanical strain treatment globally increased the chromatin accessibility of CCs, with functional implications for reprogramming potential.

As the quality of SCNT embryos is enhanced when donor cells are maintained in the G0/G1 phase (Wakayama et al., 1998), we examined the cell-cycle distributions of mechanical strain-treated CCs. No significant differences were observed between mechanical strain-treated and control CCs (Figure S2B), excluding the possibility that mechanical strain treatment increased SCNT efficiency by altering the cell cycle in CCs. Because H3K9me3 has been reported to be an epigenetic barrier to SCNT reprogramming (Liu et al., 2016; Matoba et al., 2014), we further investigated the H3K9me3 profiles of mechanical strain-treated and control CCs by performing H3K9me3 chromatin immunoprecipitation (ChIP)-seq (Table S1). The percentages of H3K9me3-enriched loci were similar between mechanical strain-treated and control CCs (2.34% and 2.68%, respectively), consistent with the comparable H3K9me3

immunofluorescence staining in the two samples (Figures S2C and S2D), indicating that H3K9me3 signals were not dramatically reduced by mechanical strain treatment.

YAP contributed to the mechanical strain-induced increase in chromatin accessibility in CCs

Since YAP is known to modulate transcription in response to mechanical signals (Yu and Guan, 2013), and since TEAD interacts with YAP to mediate YAP function (Zhao et al., 2008), probably by cooperating with AP-1 (Stein et al., 2015), we next investigated whether YAP is responsible for the increase in chromatin accessibility in mechanical strain-treated CCs. We added Super-TDU, a competitive inhibitor of the YAP-TEAD interaction (Jiao et al., 2014), to CCs prior to the mechanical strain treatment to dissect the chromatin function of YAP mediated by TEAD (Figure S3A). The DNase-TUNEL assay revealed significantly weaker fluorescence signals when Super-TDU was added than when CCs were treated with mechanical strain alone (Figures 3A and 3B). We further performed ATAC-seq in Super-TDU-supplemented mechanical strain-treated and control CCs (Table S1). While the addition of Super-TDU did not strongly influence the number of accessible chromatin regions in control CCs (38,228 and 39,376 without and with Super-TDU added, respectively), the ATAC-seq signals at accessible regions were slightly attenuated upon Super-TDU addition (Figure S3B). The number of accessible chromatin regions in mechanical strain-treated CCs dramatically decreased upon Super-TDU addition (92,419 and 68,394 without and with Super-TDU added, respectively). For mechanical strain-treated CCs, the decrease in accessible chromatin regions upon Super-TDU treatment mainly occurred at the gained accessible chromatin regions (Figures 3C, S3C, and S3D). We further divided the gained accessible chromatin regions into those that reverted to inaccessibility ("reinaccessible") and those that remained accessible upon Super-TDU treatment and performed motif analysis on both groups (see experimental procedures for details). The motifs of AP-1 factors (FOSB, FOSL2, JUN, FOSL1, JUNB, JUND, and FOS) were highly enriched in the group of reinaccessible regions (Figures 3D and S3E; Table S2), suggesting the involvement of YAP in the establishment and maintenance of chromatin accessibility in those regions, as the AP-1 motif was reported to be enriched in YAP ChIP-seq peaks (Stein et al., 2015). The motifs of nuclear receptors (STF1, NR5A2, and ERR2) showed the highest enrichment in the group of remained-accessible regions (Figure 3E). Our results indicated that the YAP-TEAD interaction is responsible for the establishment of approximately half of the chromatin regions that gained accessibility upon mechanical strain treatment, while additional factors may contribute to the remaining regions.

ms-SCNT embryos rescued the dysregulation of genome activation

We next investigated the transcriptomes of ms-SCNT embryos to understand the mechanisms underlying the improvement in efficiency. Because embryonic genome activation (EGA), the major phase of which occurs at the late 2-cell stage, is critical for the development of SCNT embryos (Matoba and Zhang, 2018), we performed RNA-seq at four stages covering the whole process of EGA (i.e., late 1 cell, early 2 cell, late 2 cell, and 4 cell) in SCNT and ms-SCNT embryos (Table S1). Compared with normal embryos, SCNT embryos exhibited 2,865 significantly downregulated genes across the EGA process, whereas only 1,142 genes were upregulated (Figure 4A), indicating that downregulation is the major type of transcriptional dysregulation in SCNT embryos. In contrast to SCNT embryos, ms-SCNT embryos showed a much smaller difference in transcriptional profile than normal embryos, with 1,298 and 598 down- and upregulated genes, respectively (Figure 4A; Table S3), suggesting the compensation of transcriptional dysregulation in ms-SCNT embryos, especially for those genes that were downregulated in SCNT embryos. Among the 2,865 genes downregulated in SCNT embryos, 35.5% displayed repaired transcription levels in ms-SCNT embryos (Figure 4B), and those repaired downregulated genes were functionally enriched in cell-cycle and blastocyst formation (Figure S4A). Our results suggested that using mechanical strain-treated CCs could partially repair the transcriptional dysregulation in SCNT embryos.

To further investigate the improvement of EGA in ms-SCNT embryos, we focused on the transcriptional patterns of 1,946 nonmaternally loaded EGA genes (see experimental procedures for details). These genes displayed a clear trend of transcription-level elevation from the late 2-cell stage in normal embryos (Figure 4C; Table S4). The transcription patterns of those EGA genes in normal embryos were more similar to those in ms-SCNT embryos than those in SCNT embryos (Figures 4C and S4B), suggesting that ms-SCNT embryos repaired the dysregulation of some EGA genes. 24 EGA genes were significantly upregulated at the late 2-cell stage in ms-SCNT embryos compared with SCNT embryos, while none of the EGA genes were significantly downregulated (Figure 4D); moreover, those 24 genes displayed similar expression patterns between ms-SCNT embryos and normal embryos (Figure 4E). Those transcriptional activation-repaired EGA genes included *Pim3*, *Pex10*, and *Sox4*, which were reported to be important for pluripotency or embryo development (Aksoy et al., 2007; Bhattaram et al., 2010; Hanson et al., 2014; Schilham et al., 1996), suggesting that the compensation of the dysregulation of some EGA genes might contribute to the improved efficiency

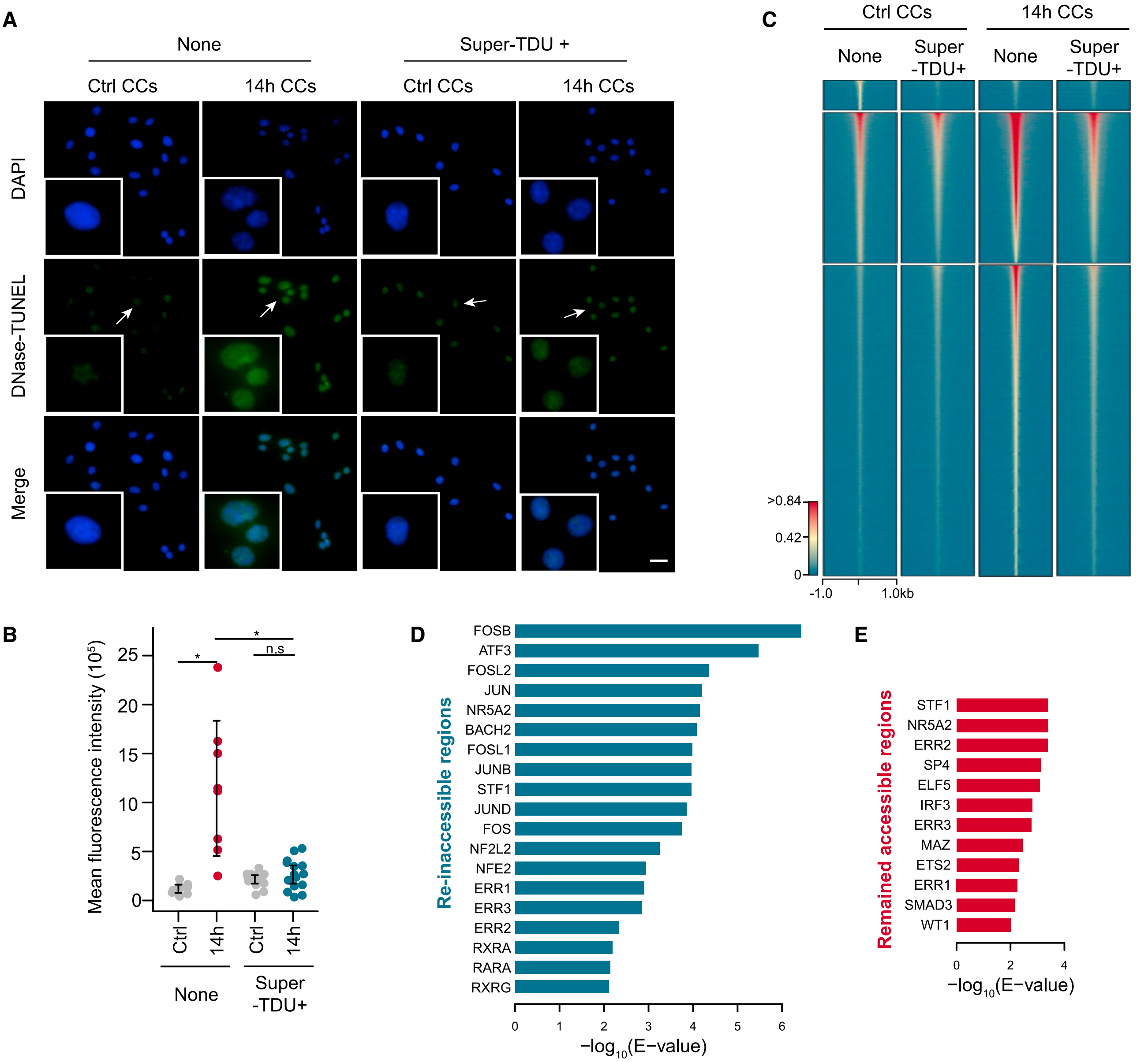

Figure 3. YAP contributed to the mechanical strain-induced chromatin accessibility gain of CCs

(A) DNase-TUNEL assay of the control and 14 h mechanical strain-treated CCs with or without super-TDU (n = 3). Scale bar, 20 μm.

(B) The quantification of positive TUNEL signals in samples with or without DNase treatment. $^{*}p < 0.05$.

(C) Heatmap of normalized ATAC-seq signals around peak summits demonstrated a genome-wide increase in chromatin accessibility induced by mechanical strain, while ATAC-seq signals decreased upon super-TDU treatment (n = 2). Heatmap clustering was ordered from strongest to weakest signal.

(D and E) Bar plot showing the significance of *de novo* motif discovery in reinaccessible regions (D) and remained-accessible regions (E).

of ms-SCNT embryos. Among the 24 genes, 8 gained accessible chromatin regions around their transcription start sites (TSSs) in CCs (Figures 4F and S4C). We further predicted that chromatin regulators including KMT2C/D, BRD4, SMAD3, and TBP have the potential to bind accessible chromatin regions around their TSSs (see experimental procedures for details); these regulators have also been reported to be functionally important during reprogramming or pluripotency maintenance (Di Micco et al., 2014; Dunn et al., 2004; Veenstra et al., 2000; Wang et al., 2016) (Figures 4F and S4D), suggesting that mechanical strain treatment might contribute to the binding of reprogramming factors in ms-SCNT embryos to repair the dysregulation of genome activation.

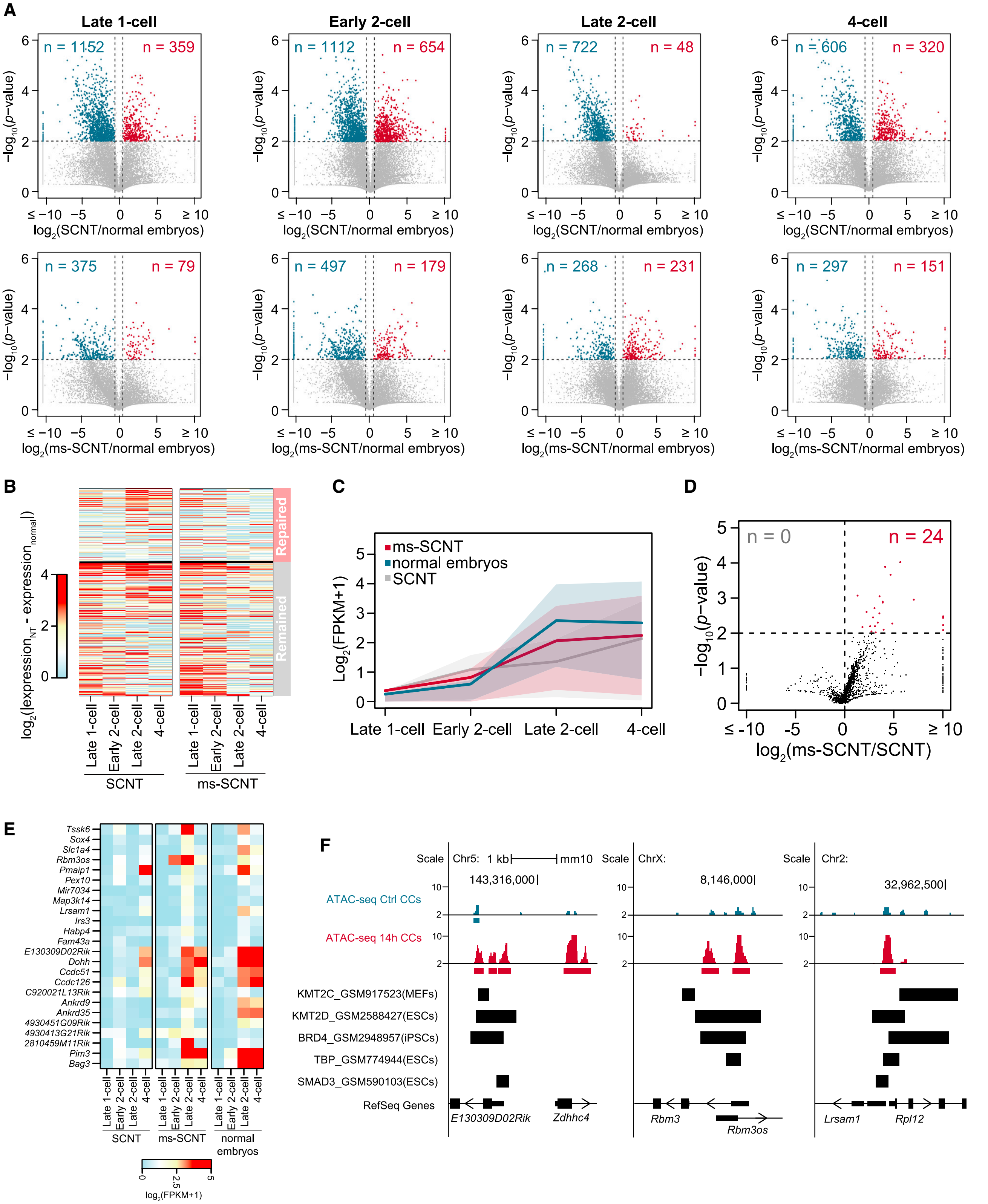

(legend on next page)

DISCUSSION

This study utilized mechanical strain to improve SCNT reprogramming efficiency, which emphasized the relationship between the quantifiable modulation of mechanical properties and cell fate transition. We further demonstrated that the improvement was achieved via a mechanical strain-induced increase in chromatin accessibility, which indicated that chromatin remodeling can play a mediating role in linking the response to mechanical treatment and cell fate reprogramming potential. Our results are consistent with recent studies, which reported that mechanical force from matrix stiffness can promote cell transformation to a transient state, accompanied by an increase in chromatin accessibility (Stowers et al., 2019; Walker et al., 2021). To the best of our knowledge, our study is the first report describing the modulation of mechanical properties to improve SCNT reprogramming efficiency. In the future, it will be worthwhile to combine the mechanical strain treatment with other known approaches to investigate whether multifactorial effects can further improve the SCNT reprogramming efficiency.

Recent studies indicated that the level of H3K9me3, a well-characterized heterochromatin mark, can be drastically decreased upon mechanical treatment (Le et al., 2016; Nava et al., 2020) and that erasing H3K9me3 can make chromatin more active in response to force (Sun et al., 2020). However, in our study, we observed that the level of H3K9me3 did not decrease globally upon mechanical strain treatment, suggesting the complexity of multifaceted chromatin responses to mechanical treatment. The diversity of chromatin responses upon mechanical treatments may be due to differences in the mechanical treatment type, strength, and duration used, together with cell type.

Our study clearly demonstrated the usefulness of cell state transition signature analysis (Zhu et al., 2017) in predicting cell state transition potential. Those signatures were derived from time-series gene expression data during cell state transitions, and at least some genes in each signature can reflect the features of intermediate states of a given transition process, which are usually transient but can be informative for revealing cell fate determination potential. Cell state transition signature analysis can be applied in future studies to facilitate the identification of ideal conditions for inducing cell fate transition.

EXPERIMENTAL PROCEDURES

Resource availability

Corresponding author

Further information and requests for resources and reagents should be addressed to the lead contact, Yong Zhang (yzhang@tongji.edu.cn).

Materials availability

No unique reagents were generated in this study.

Data and code availability

All ATAC-seq, ChIP-seq, and RNA-seq datasets generated in this study are summarized in Table S1 and have been deposited in the Genome Sequence Archive (https://bigd.big.ac.cn/gsa/) under GSA: CRA005906.

The details of experimental procedures are provided in the supplemental information.

SUPPLEMENTAL INFORMATION

Supplemental information can be found online at https://doi.org/10.1016/j.stemcr.2023.02.007.

AUTHOR CONTRIBUTIONS

Y.Z. conceived the project; Y.C. and Y.Z. designed the research strategy; Y.C., C.L., R.X., S.Z., and Z.H. performed experiments under the supervision of S.G., Y.S., J.L., and X.L.; C.Z., Y.H., and Y.X. performed computational analysis; Y.C. and Y.Z. wrote the manuscript.

ACKNOWLEDGMENTS

We thank Guifen Liu, Qi Wang, Xiaolan Chen, Hui Yang, Yi Eve Sun, Jiping Liu, and Xingming Zhao for their help. This work was supported by the National Natural Science Foundation of

Figure 4. Genome activation dysregulation is repaired in ms-SCNT embryos

(A) Volcano plot of gene expression levels compared between SCNT and normal *in vivo* fertilized embryos (top) and between ms-SCNT and normal *in vivo* fertilized embryos (bottom) at the late 1-cell, early 2-cell, late 2-cell, and 4-cell stages ($n \geq 2$). Significantly differentially expressed genes are highlighted in blue and red.

(B) The heatmap demonstrates the expression level differences between normal *in vivo* fertilized embryos in SCNT and ms-SCNT embryos for the 2,865 genes downregulated in SCNT embryos.

(C) The line plot shows the expression level of nonmaternally loaded EGA genes in SCNT, ms-SCNT, and normal embryos during early development.

(D) Volcano plot of gene expression levels of nonmaternally loaded EGA genes compared between SCNT and ms-SCNT embryos at the late 2-cell stage.

(E) Heatmap displaying the expression levels of 24 nonmaternally loaded EGA genes.

(F) Genome browser snapshots of representative promoter regions of nonmaternally loaded EGA genes. The tracks include the chromatin regulator binding sites predicted by Cistrome DB Toolkit significance analysis.

China (32030022 and 31970642 to Y.Z.; 31721003 to S.G.; 82061130222 to Y.S.; and 31900491 to C.Z.); the National Key Research and Development Program of China (2021YFA1302500 to Y.Z. and 2020YFA0112500 to C.L.); the China Postdoctoral Science Foundation (2021M702488 to Y.C.); Shanghai Super Postdoctoral Program of Shanghai Talent Programs (2020383 to Y.C.); Peak Disciplines (Type IV) of Institutions of Higher Learning in Shanghai (Y.Z. and S.G.); Shanghai Academic Leader of Science and Technology Innovation Action Plan (20XD1424000 to Y.S.); and the Shanghai Experimental Animal Research Project of Science and Technology Innovation Action Plan (201409006400 to Y.S.).

CONFLICT OF INTERESTS

The authors declare no competing interests.

Received: August 2, 2022
Revised: February 19, 2023
Accepted: February 21, 2023
Published: March 23, 2023

REFERENCES

Aksoy, I., Sakabedoyan, C., Bourillot, P.Y., Malashicheva, A.B., Mancip, J., Knoblauch, K., Afanassieff, M., and Savatier, P. (2007). Self-renewal of murine embryonic stem cells is supported by the serine/threonine kinases Pim-1 and Pim-3. Stem Cell *25*, 2996–3004.

Bhattaram, P., Penzo-Mendez, A., Sock, E., Colmenares, C., Kaneko, K.J., Vassilev, A., Depamphilis, M.L., Wegner, M., and Lefebvre, V. (2010). Organogenesis relies on SoxC transcription factors for the survival of neural and mesenchymal progenitors. Nat. Commun. *1*, 9.

Chan, C.J., Costanzo, M., Ruiz-Herrero, T., Mönke, G., Petrie, R.J., Bergert, M., Diz-Muñoz, A., Mahadevan, L., and Hiiragi, T. (2019). Hydraulic control of mammalian embryo size and cell fate. Nature *571*, 112–116.

Chowdhury, F., Na, S., Li, D., Poh, Y.C., Tanaka, T.S., Wang, F., and Wang, N. (2010). Material properties of the cell dictate stress-induced spreading and differentiation in embryonic stem cells. Nat. Mater. *9*, 82–88.

Connelly, J.T., Gautrot, J.E., Trappmann, B., Tan, D.W.M., Donati, G., Huck, W.T.S., and Watt, F.M. (2010). Actin and serum response factor transduce physical cues from the microenvironment to regulate epidermal stem cell fate decisions. Nat. Cell Biol. *12*, 711–718.

Di Micco, R., Fontanals-Cirera, B., Low, V., Ntziachristos, P., Yuen, S.K., Lovell, C.D., Dolgalev, I., Yonekubo, Y., Zhang, G., Rusinova, E., et al. (2014). Control of embryonic stem cell identity by BRD4-dependent transcriptional elongation of super-enhancer-associated pluripotency genes. Cell Rep. *9*, 234–247.

Dunn, N.R., Vincent, S.D., Oxburgh, L., Robertson, E.J., and Bikoff, E.K. (2004). Combinatorial activities of Smad2 and Smad3 regulate mesoderm formation and patterning in the mouse embryo. Development *131*, 1717–1728.

Engler, A.J., Sen, S., Sweeney, H.L., and Discher, D.E. (2006). Matrix elasticity directs stem cell lineage specification. Cell *126*, 677–689.

Gilbert, P.M., Havenstrite, K.L., Magnusson, K.E.G., Sacco, A., Leonardi, N.A., Kraft, P., Nguyen, N.K., Thrun, S., Lutolf, M.P., and Blau, H.M. (2010). Substrate elasticity regulates skeletal muscle stem cell self-renewal in culture. Science *329*, 1078–1081.

Graf, T., and Enver, T. (2009). Forcing cells to change lineages. Nature *462*, 587–594.

Hanson, M.G., Fregoso, V.L., Vrana, J.D., Tucker, C.L., and Niswander, L.A. (2014). Peripheral nervous system defects in a mouse model for peroxisomal biogenesis disorders. Dev. Biol. *395*, 84–95.

Jiao, S., Wang, H., Shi, Z., Dong, A., Zhang, W., Song, X., He, F., Wang, Y., Zhang, Z., Wang, W., et al. (2014). A peptide mimicking VGLL4 function acts as a YAP antagonist therapy against gastric cancer. Cancer Cell *25*, 166–180.

Le, H.Q., Ghatak, S., Yeung, C.Y.C., Tellkamp, F., Günschmann, C., Dieterich, C., Yeroslaviz, A., Habermann, B., Pombo, A., Niessen, C.M., and Wickström, S.A. (2016). Mechanical regulation of transcription controls Polycomb-mediated gene silencing during lineage commitment. Nat. Cell Biol. *18*, 864–875.

Liu, W., Liu, X., Wang, C., Gao, Y., Gao, R., Kou, X., Zhao, Y., Li, J., Wu, Y., Xiu, W., et al. (2016). Identification of key factors conquering developmental arrest of somatic cell cloned embryos by combining embryo biopsy and single-cell sequencing. Cell Discov. *2*, 16010.

Matoba, S., Liu, Y., Lu, F., Iwabuchi, K.A., Shen, L., Inoue, A., and Zhang, Y. (2014). Embryonic development following somatic cell nuclear transfer impeded by persisting histone methylation. Cell *159*, 884–895.

Matoba, S., and Zhang, Y. (2018). Somatic cell nuclear transfer reprogramming: mechanisms and applications. Cell Stem Cell *23*, 471–485.

McBeath, R., Pirone, D.M., Nelson, C.M., Bhadriraju, K., and Chen, C.S. (2004). Cell shape, cytoskeletal tension, and RhoA regulate stem cell lineage commitment. Dev. Cell *6*, 483–495.

Nava, M.M., Miroshnikova, Y.A., Biggs, L.C., Whitefield, D.B., Metge, F., Boucas, J., Vihinen, H., Jokitalo, E., Li, X., García Arcos, J.M., et al. (2020). Heterochromatin-driven nuclear softening protects the genome against mechanical stress-induced damage. Cell *181*, 800–817.e22.

Schilham, M.W., Oosterwegel, M.A., Moerer, P., Ya, J., de Boer, P.A., van de Wetering, M., Verbeek, S., Lamers, W.H., Kruisbeek, A.M., Cumano, A., and Clevers, H. (1996). Defects in cardiac outflow tract formation and pro-B-lymphocyte expansion in mice lacking Sox-4. Nature *380*, 711–714.

Stein, C., Bardet, A.F., Roma, G., Bergling, S., Clay, I., Ruchti, A., Agarinis, C., Schmelzle, T., Bouwmeester, T., Schübeler, D., and Bauer, A. (2015). YAP1 exerts its transcriptional control via TEAD-mediated activation of enhancers. PLoS Genet. *11*, e1005465.

Stowers, R.S., Shcherbina, A., Israeli, J., Gruber, J.J., Chang, J., Nam, S., Rabiee, A., Teruel, M.N., Snyder, M.P., Kundaje, A., and Chaudhuri, O. (2019). Matrix stiffness induces a tumorigenic phenotype in mammary epithelium through changes in chromatin accessibility. Nat. Biomed. Eng. *3*, 1009–1019.

Sun, J., Chen, J., Mohagheghian, E., and Wang, N. (2020). Force-induced gene up-regulation does not follow the weak power law but depends on H3K9 demethylation. Sci. Adv. *6*, eaay9095.

Tajik, A., Zhang, Y., Wei, F., Sun, J., Jia, Q., Zhou, W., Singh, R., Khanna, N., Belmont, A.S., and Wang, N. (2016). Transcription upregulation via force-induced direct stretching of chromatin. Nat. Mater. *15*, 1287–1296.

Veenstra, G.J., Weeks, D.L., and Wolffe, A.P. (2000). Distinct roles for TBP and TBP-like factor in early embryonic gene transcription in Xenopus. Science *290*, 2312–2315.

Wakayama, T., Perry, A.C., Zuccotti, M., Johnson, K.R., and Yanagimachi, R. (1998). Full-term development of mice from enucleated oocytes injected with cumulus cell nuclei. Nature *394*, 369–374.

Walker, C.J., Crocini, C., Ramirez, D., Killaars, A.R., Grim, J.C., Aguado, B.A., Clark, K., Allen, M.A., Dowell, R.D., Leinwand, L.A., and Anseth, K.S. (2021). Nuclear mechanosensing drives chromatin remodelling in persistently activated fibroblasts. Nat. Biomed. Eng. *5*, 1517–1518.

Wang, C., Lee, J.E., Lai, B., Macfarlan, T.S., Xu, S., Zhuang, L., Liu, C., Peng, W., and Ge, K. (2016). Enhancer priming by H3K4 methyltransferase MLL4 controls cell fate transition. Proc. Natl. Acad. Sci. USA *113*, 11871–11876.

Yu, F.X., and Guan, K.L. (2013). The Hippo pathway: regulators and regulations. Genes Dev. *27*, 355–371.

Zhao, B., Ye, X., Yu, J., Li, L., Li, W., Li, S., Yu, J., Lin, J.D., Wang, C.Y., Chinnaiyan, A.M., et al. (2008). TEAD mediates YAP-dependent gene induction and growth control. Genes Dev. *22*, 1962–1971.

Zhu, G., Yang, H., Chen, X., Wu, J., Zhang, Y., and Zhao, X.M. (2017). CSTEA: a webserver for the cell state transition expression atlas. Nucleic Acids Res. *45*, W103–W108.

Stem Cell Reports, Volume *18*

Supplemental Information

Mechanical strain treatment improves nuclear transfer reprogramming efficiency by enhancing chromatin accessibility

Yujie Chen, Ruimin Xu, Shuang Zhou, Chengchen Zhao, Ziyue Hu, Yuwei Hua, Yanhong Xiong, Xiaoyu Liu, Junhong Lü, Yao Sun, Chong Li, Shaorong Gao, and Yong Zhang

SUPPLEMENTARY FIGURE

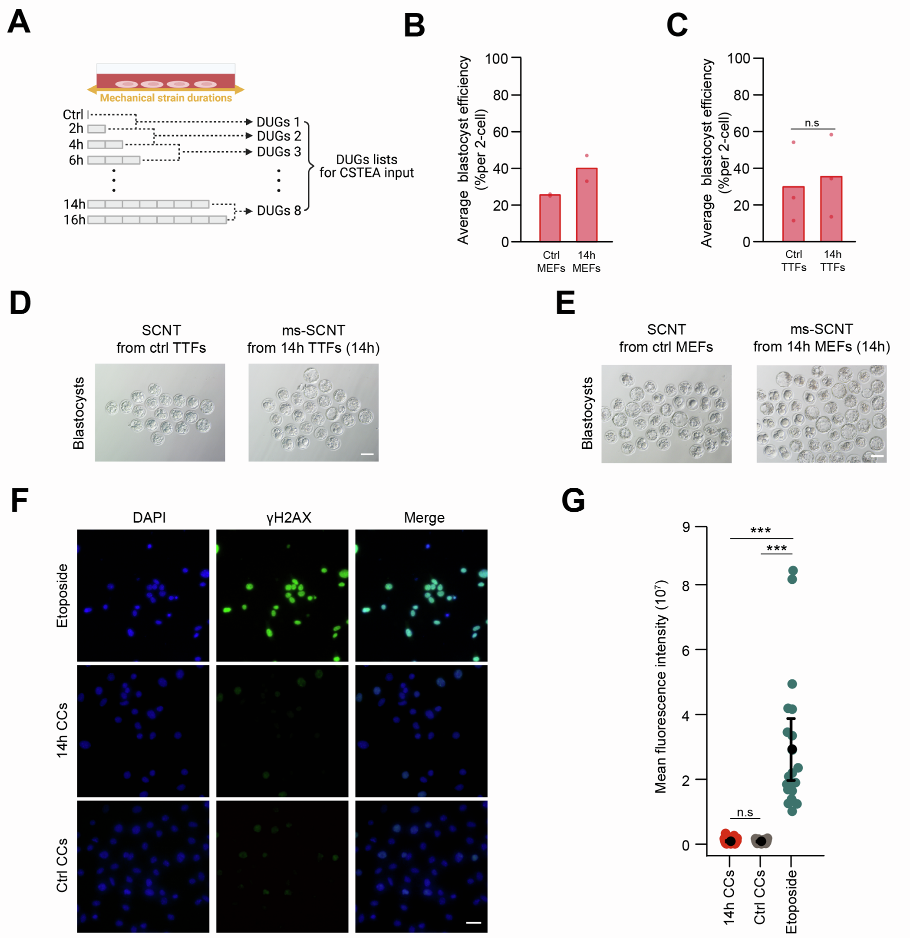

Supplementary Figure 1. Mechanical strain treatment improved the efficiency of SCNT using CCs and other cell types. (A) The workflow for identifying differentially upregulated genes (DUGs) for signature querying in CSTEA (n≥2), and this figure was created with BioRender (https://app.biorender.com). (B, C) The blastocyst developmental efficiency of 14 h mechanical strain-treated mouse embryonic fibroblast cell (MEF) (n=2) (B) and tail-tip fibroblast cell (TTF) (n=3) -derived ms-SCNT embryos (C). (D, E) The blastocyst phenotype of MEFs (D) and TTFs (e) 14 h ms-SCNT blastocyst phenotype. Scale bar, 100 μm. (F) Representative images of γH2AX staining in control and 14 h mechanical strain-treated CCs, with Etoposide-treated sample as the positive control. Scale bar, 50 μm (n=3). (G) Quantification of signals in γH2AX staining. Significant differences, ***$p < 0.001$.

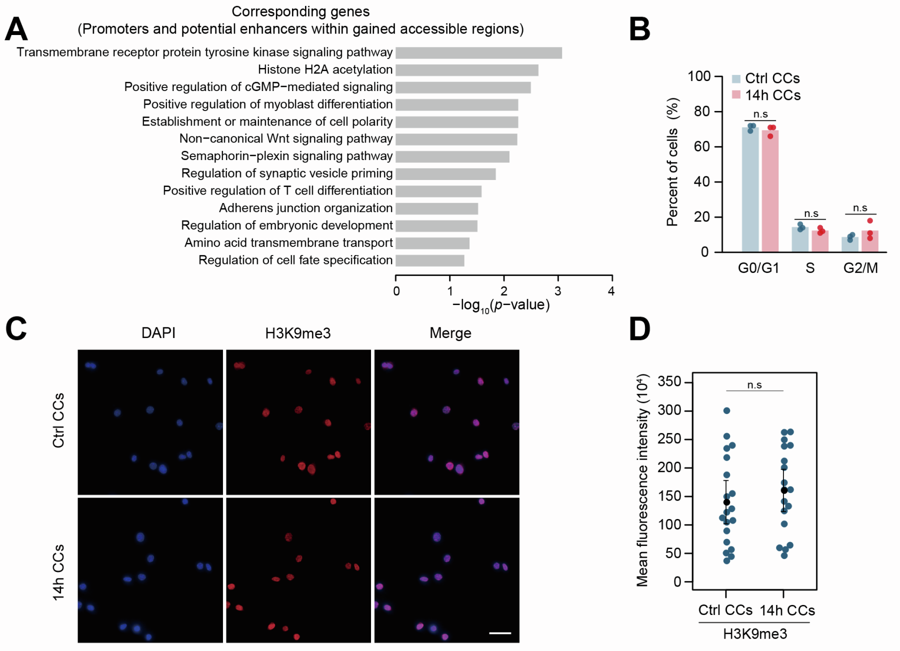

Supplementary Figure 2. Mechanical strain treatment did not globally decrease the H3K9me3 signal in CCs. (A) GO enrichment analysis of the genes whose promoters and potential enhancers overlapped with gained accessibility regions. The *p* value was calculated with Fisher's exact test. (B) The bar plot demonstrates the cell cycle distribution in control and 14 h mechanical strain-treated CCs (n=3). (C) Representative images of H3K9me3 staining in control and 14 h mechanical strain-treated CCs (n=3). Scale bar, 50 μm. (D) The quantification of positive signals in H3K9me3 staining.

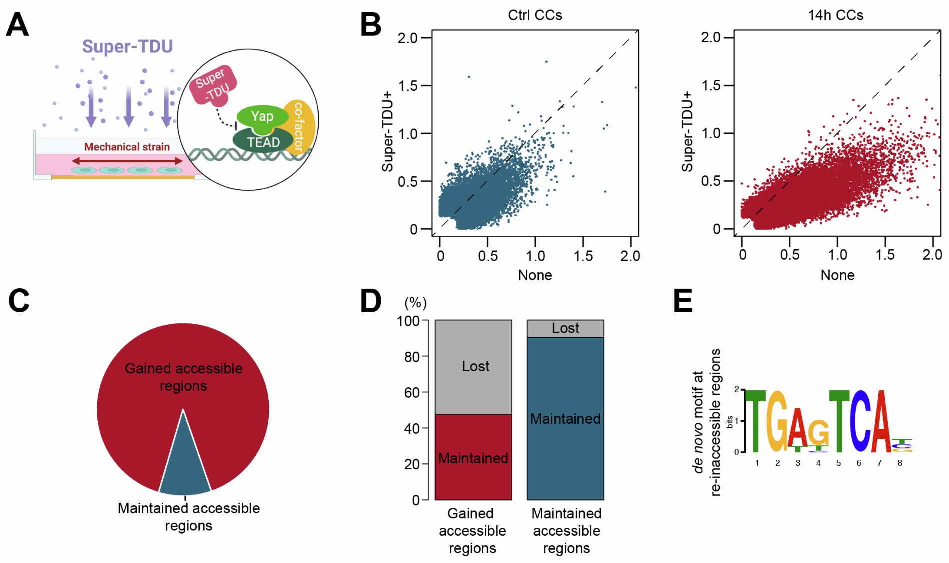

Supplementary Figure 3. YAP contributed to mechanical strain-induced increases in chromatin accessibility. (A) Schematic of the super-TDU function, and this figure was created with BioRender (https://app.biorender.com). (B) Scatterplots of normalized ATAC-seq signals of control CCs with and without Super-TDU (left) and mechanical strain-treated CCs with and without Super-TDU (right) (n=2). (C) Pie chart showing the proportion of original categories for the regions losing accessibility upon super-TDU treatment. (D) The stacked bar plot shows the proportion of regions with maintained and lost accessibility upon super-TDU treatment. (E) The sequence logo of the *de novo* discovered motif in re-inaccessible regions.

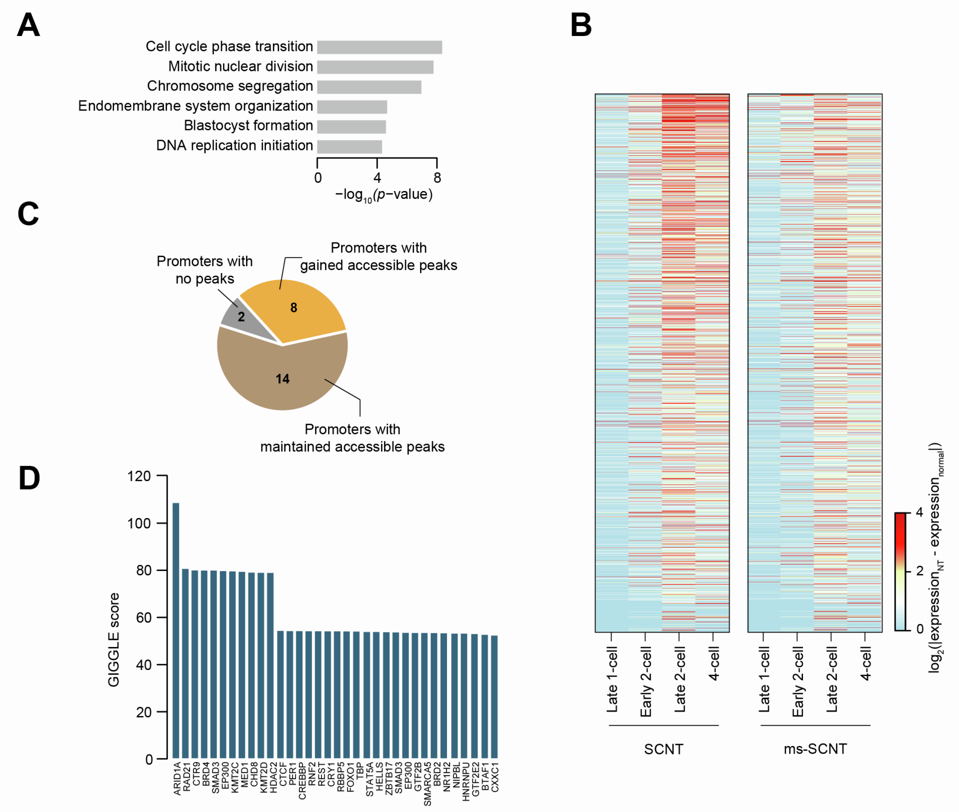

Supplementary Figure 4. Regions with mechanical strain-induced gains in accessibility affected ms-SCNT embryo development. (A) GO enrichment analysis of genes with repaired downregulation. The *p* value was calculated by Fisher's exact test. (B) The heatmap demonstrates the expression level differences of nonmaternally loaded EGA genes between normal *in vivo* fertilized embryos and ms-SCNT embryos (n≥2). (C) Pie chart demonstrating the proportion of 24 nonmaternally loaded EGA genes with gained or maintained promoter accessibility in mechanical strain-treated CCs. (D) Bar plot demonstrating the chromatin regulators predicted by the Cistrome DB Toolkit.

Table S1: List of all performed datasets including RNA-seq, ATAC-seq and ChIP-seq

This table is provided as a separate Excel sheet.

Table S2: The E-values of AP-1-related factor motifs in re-inaccessible regions, related to Figure3.

Factors	E-value
FOSB	3.75×10^{-7}
ATF3	3.40×10^{-6}
FOSL2	4.48×10^{-5}
JUN	6.24×10^{-5}

Table S3: Table of the overlap between downregulated genes in SCNT and ms-SCNT embryos, related to Figure 4

Stages	Down regulated genes			
	SCNT	ms-SCNT	Overlapped	*p*-value
Late 1-cell	1152	375	243	2.92×10^{-232}
Early 2-cell	1112	497	300	3.43×10^{-281}
Late 2-cell	722	268	123	4.52×10^{-116}
4-cell	606	297	141	5.47×10^{-148}

Table S4: Table showing the statistical significance of EGA genes' expression differences between SCNT / ms-SCNT and normal embryos. The *p* value was calculated using the paired Wilcoxon test, related to Figure 4

	Late 1-cell	Early 2-cell	Late 2-cell	4-cell
SCNT vs. normal	1.11×10^{-4}	2.44×10^{-107}	1.45×10^{-232}	2.16×10^{-50}
ms-SCNT vs. normal	7.74×10^{-4}	6.62×10^{-16}	1.08×10^{-72}	1.45×10^{-51}

SUPPLEMENTAL EXPERIMENTAL PROCEDURE

Mice

BDF1 (C57BL/6 × DBA/2) and ICR mice were housed in the specific pathogen-free-grade animal facility of Tongji University. All mouse maintenance and experiments were performed following the Tongji University Laboratory Animals Procedures. Cells were isolated from our mouse strain. Briefly, cumulus cells (CCs) and tail-tip fibroblast cells (TTFs) were isolated from BDF1 mice, and mouse embryonic fibroblast cells (MEFs) were isolated and cultured from ICR mouse embryos at 13.5 dpc.

Cell culture and mechanical strain treatment

Approximately 1.2 × 10^5 cells per well were seeded on a 6-well BioFlex® culture plate with a silicon elastomer membrane, and the cells were cultured in medium (Sigma) supplemented with glutamine (Millipore) and penicillin–streptomycin (Gibco). Cells were seeded on plates for at least 2 h to allow cells to attach stably before being stretched, and changing the medium before stretching was performed as recommended. Then, the cells were stretched under cyclic mechanical strain using the Flexcell Tension System (FX5000T; Flexcell International Corporation) at 7.5% and a frequency of 0.5 Hz for all durations. To interfere with the YAP-TEAD interaction, cells were exposed to 9.5 μmol Super-TDU (Selleck) before stretching began.

SCNT and embryo culture

SCNT was carried out according to the procedures described in our previous work(Gao et al., 2018). Briefly, 8~10-week-old female BDF1 mice were injected with 5 units of pregnant mare serum gonadotropin (PMSG), followed after 48 h by 6 units of human chorionic gonadotropin (hCG, San-Sheng Pharmaceutical). MII oocytes were isolated from the dissected oviducts 13 h later and transferred into prewarmed Chatot–Ziomek–Bavister medium (CZB). CCs were collected into 1.5 mL tubes from the oocyte-cumulus complex after hyaluronidase (Sigma) treatment at 37 °C in 5% CO_2, followed by two washes with HEPES-CZB. All MII oocytes were enucleated in HEPES-CZB containing 5 μg/mL cytochalasin B (Sigma) and kept at 37 °C and 5% CO_2 until SCNT. The nuclei of mechanical strain-treated and control donor cells were injected into enucleated MII oocytes with a piezo-driven micromanipulator (Eppendorf). The reconstructed embryos were cultured in CZB for 1 h and activated in calcium-free CZB containing 1% $SrCl_2$ for 5 h. All reconstructed embryos were transferred to G-1 PLUS medium (Vitrolife) for further culture during preimplantation development.

Immunofluorescence staining in embryos and cells

For preimplantation embryo immunofluorescence staining, blastocysts were fixed in 4% paraformaldehyde for 1 h at room temperature and washed twice for 15 min with 0.5% BSA-PBS. All fixed embryos were incubated in anti-OCT4 (Santa Cruz Biotechnology) and anti-CDX2 (Abcam) antibody at 4 °C overnight in 0.5% BSA-PBS containing 0.1% Triton and washed twice for 15 min with 0.5% BSA-PBS at room temperature. The incubation with conjugated secondary antibody (Invitrogen) was performed in 0.5% BSA-PBS at room temperature for 1 h; then, the cells were washed twice, incubated with DAPI for 15 min at room temperature, and imaged. For cell immunofluorescence staining, cells were incubated in anti-

H3K9me3 (Active Motif) or γH2AX (Beyotime) antibody, and the positive control in γH2AX immunofluorescence staining was treated with the medium containing 100 μM etoposide (Selleck) for 1.5 h. All washing steps and DAPI incubation were performed for 5 min, and the other procedures were as described above.

DnaseI-TUNEL

Before DNase I treatment, cells seeded in BioFlex® culture plates were washed twice with 1X calcium-free PBS and incubated with PBS containing 0.2% Triton X-100 for 5 min followed by two washes. Cells were incubated with 200 μL of fresh DNase I buffer (40 mM pH 7.9 Tris-HCl, 10 mM NaCl, 6 mM $MgCl_2$, 10 mM $CaCl_2$) for 5 min and then replaced with buffer containing 5.5 units/mL DNase I (Roche) for 10 min of incubation at room temperature. After DNase I treatment, the cells were fixed immediately with 1× paraformaldehyde buffer (Sangon Biotech) for 10 min and washed twice with PBS. The cells were preincubated at room temperature for 5~10 min with 200 μL equilibration buffer (25 mM pH 7.9 Tris-HCl, 20 mM potassium cacodylate, 0.2 mM DTT, and 0.25 mg/mL BSA), and then the TDT reaction buffer was replaced with 7 μL nucleotide mix, 1.5 μL rTDT (Promega) and 63 μL equilibration buffer at 37 °C for 1 h. The bottom membranes around the edges of the wells were cut out and then washed for 5 min by immersion in PBS. The membranes were then immersed in 20 mL 2× SSC (Promega) for 15 min, washed 3 times with PBS, incubated with DAPI for 5 min and imaged.

ATAC-seq

To prepare the nuclei, 1 × 10^5 cells were harvested per BioFlex® culture plate well, and the cells were spun down at 500 × g for 5 min, followed by washing with 200 μL of cold 1× PBS. Cells were lysed in 50 μL of fresh and cold lysis buffer (10 mM pH 7.4 Tris-HCl, 10 mM NaCl, 3 mM $MgCl_2$, 0.1% IGEPAL CA-630), gently pipetted several times, and then placed on ice for 10 min. The samples were immediately spun in a refrigerated centrifuge at 500 × g for 5 min, the supernatants were carefully removed, and the precipitates were kept on ice. For the following fragmentation step, the precipitates were resuspended in 20 μL fragment master mix (4 μL 5× TTBL, 5 μL TTE mix (Vazyme), 11 μL nuclease-free water) and gently pipetted several times on ice; then, the reaction was carried out at 37 °C for 30 min. The reaction was directly stopped by adding 5 μL of 5× TS (Vazyme) and transferring the reaction to room temperature for 5 min. Then, and the samples were purified with AMPure XP beads. To generate the amplified library, the primers and the master mix (Vazyme) were mixed with the fragments, amplified for a total of 8-10 cycles, and purified with 1.2× AMPure XP beads.

Native ChIP-seq

A total of 1 × 10^5 cells were washed twice with 1× PBS and resuspended in 20 μL cold nuclear extraction buffer (10 mM pH 8.5 Tris-HCl, 140 mM NaCl, 5 mM $MgCl_2$, 0.6% NP-40, 0.1 mM phenylmethylsulfonyl fluoride, and 1 × protease inhibitor cocktail (PIC)). thoroughly mixed, and incubated on ice for 1 min. The cells were then spun in a refrigerated centrifuge at 4000 rpm for 5 min, and carefully absorbed supernatants away. MNase (NEB) was diluted to 10 U/μL with MNase dilution buffer (10 mM pH 7.5 Tris-HCl, 50 mM NaCl, 1 mM ethylenediaminetetraacetic acid, 50% glycerin), and MNase master buffer was prepared (10 mM pH 7.5 Tris-HCl, 1 mM $CaCl_2$); then, 40 μL of MNase master mix (5 μL MNase master buffer, 2 mM dithiothreitol, 5%

PEG6000, 3 μL 10 U/μL MNase) was used to fully resuspend the precipitates, followed by a 5 min incubation at 37 °C. A 5.5 μL aliquot of MNase stop buffer (1:4 0.5 mM ethylenediaminetetraacetic acid:nuclease-free water) was used to stop the reaction, and 5.5 μL nuclear break buffer (1% deoxycholate and 1% Triton X-100) was added to release the chromatin. Chromatin was pre-cleared with 10 μL of 1:1 protein A:protein G Dynabeads (Life Technologies) at 4 °C for 1 h. One milligram of H3K9me3 antibody (Active motif no. 39161) was pre-incubated with Dynabeads, and the antibody-beads complex was mixed with the chromatin sample and incubated overnight at 4 °C. The samples were washed twice with 200 μL low-salt washing buffer (20 mM pH 8.0 Tris-HCl, 0.1% SDS, 1% Triton X-100, 2 mM ethylenediaminetetraacetic acid, 150 mM NaCl, 1 ×PIC), and twice with 200 μL high-salt washing buffer (20 mM pH 8.0 Tris-HCl, 0.1% SDS, 1% Triton X-100, 2 mM ethylenediaminetetraacetic acid, 500 mM NaCl, 1 ×PIC). IP material was eluted from the beads with 100 μL hot elution buffer (100 mM $NaHCO_3$ and 1% SDS) at 65 °C for 1.5~2 h, and the supernatants were transferred to a new tube. Then, the IP material was treated with 2.5 μL 20 mg/mL proteinase K at 55 °C for 30 min, purified with phenol chloroform, ethanol-precipitated and resuspended in 10 mM pH 8.0 Tris-HCl. DNA libraries were constructed with KAPA Hyper Prep Kit. In brief, samples were end-repaired with A-tailing, adapter ligated and amplified for 10 cycles. Libraries were purified with 1.2× AMPure XP beads.

RNA-seq

For the RNA-seq performed in CCs, in brief, cells were lysed, followed immediately by the reverse transcription process to generate first-strand cDNAs, 3' poly(A) tailing, and adapter ligation. The adapter-ligated cDNAs were amplified for 18 cycles for cDNA library construction with KAPA Hyper Prep Kit, followed by AMPure XP bead purification. For the single-embryo RNA-seq, late 1-cell, early 2-cell, late 2-cell and 4-cell reconstructed embryos were harvested. cDNA libraries were synthesized and amplified using SMARTer Ultra Low Input RNA Kit for sequencing following the manufacturer's instructions. Fragments of 0.5-5 kb were selected by 2% agarose gel, and cDNA purification was performed using a QIAquick PCR Purification Kit.

Sequencing data processing

ATAC-seq reads were mapped to the mm10 reference genome after trimming the adaptors using bowtie2 (v2.3.5.1, the parameters used were --trim-to 3:40 -x mm10)(Langmead and Salzberg, 2012), and SAMtools(Li et al., 2009) was used to remove the repetitive, lower quality (Q≤30) and mitochondrial DNA reads from the total mapped reads. Reads with lengths less than 50 base pairs (bp) were retained for subsequent analysis. To allow the comparison of data with different sequencing depths, the signals were normalized to ten million reads for each sample, and the values were further compressed into a binary format (bigWig) for downstream analysis and data visualization. Peak calling was performed using MACS (v1.4.2)(Zhang et al., 2008) with the following parameters: -g mm --keep-dup all --nomodel --shiftsize 25.

H3K9me3 ChIP-seq read mapping was carried out as previously described, and the peaks were called using MACS (v2.1.3)(Zhang et al., 2008) with parameters as follows: -g mm -p 1e-5 --broad --broad-cutoff 1e-5 --keep-dup all. The signals were normalized to twenty million reads for each sample. The CC and embryo RNA-seq reads were mapped to the mm10 reference

genome with HISAT2 (v2.1.0)(Kim et al., 2019), and StringTie (v1.3.3b)(Pertea et al., 2015) was used to quantify the transcription level of each gene in each sample as FPKM (fragments per kilobase of exon per million mapped fragments).

Prediction of mechanically induced cell fate transition potential

Given the FPKM matrix generated above, GFOLD(Feng et al., 2012) was used to perform differential expression analysis between adjacent duration-conditions, and the genes with a GFOLD value > 0.5 were considered differentially upregulated genes (DUGs). The details are provided in the Supplemental Information. The numbers of all DUGs from every paired treatment condition were counted, and the DUGs that appeared at least twice in all adjacent conditions were considered as features for subsequent analysis. Default parameters in CSTEA(Zhu et al., 2017) were used for signature querying of these DUGs, and the top five queried processes are shown. The brief description of cell state transition signature analysis of CSTEA is as follows. For each publicly available time-series gene expression data, CSTEA defined its cell state transition signature as the union of differentially expressed genes at two consecutive time points. If two or more datasets with the same origin and destination cell types were available, their signatures were combined. Given a querying gene set, its enrichment analysis was performed using Fisher's exact test against all defined cell state transition signatures. The link of CSTEA webserver is https://mai.fudan.edu.cn/cstea/.

Genome annotation

Promoters were defined as the regions ± 2 kb around the transcription start sites (TSSs) of RefSeq genes, and potential enhancers were defined as the union set of public H3K27ac ChIP-seq peaks and H3K4me1 ChIP-seq peaks in mice early embryos, excluding the promoters. We downloaded the public ChIP-seq data from Gene Express Omnibus (GEO). H3K27ac ChIP-seq data included GSE185653 (zygote, early 2-cell, late 2-cell, 4-cell), GSE207222 (zygote, early 2-cell, late 2-cell), GSE125318 (E6.5) and GSE195592 (E8.4). H3K4me1 ChIP-seq data included GSE98101 (E6.5, E7.0, E7.5), GSE94131 (E8.5, E9.5) and GSE79941 (E9.5). Peak calling was performed using MACS (v1.4.2)(Zhang et al., 2008) with the default parameters. We merged the overlapping peaks, and defined these peaks as potential enhancers in mice. If a potential enhancer is located within ± 10 kb of a gene's TSS, it was defined as the gene's potential enhancer. If a gained accessible chromatin region was located within the promoter or potential enhancer of a gene, that gene was regarded as corresponding to the gained accessible chromatin region.

Functional annotation analysis

Functional annotation was performed using the Database for Annotation, Visualization and Integrated Discovery (DAVID) Bioinformatics Resource 6.8(Huang et al., 2009). Gene Ontology terms for each functional cluster were summarized to a representative term, and *p* values were plotted to show the significance.

Nonmaternally loaded EGA genes

The list of 1,946 nonmaternally loaded EGA genes was defined as follows: high transcription level (FPKM > 5) in 2-cell stage embryos and low transcription level (FPKM < 1) in MII oocytes. Public data (GSE71434) were used for this analysis.

Motif analysis

Motif analysis was performed using MEME suite tools (v5.0.5)(Bailey et al., 2015) against the genome sequence of the given ATAC-seq peak regions (peak summits ± 100 bp) with the following parameters: -maxsize 20000000 -mod zoops -nmotifs 30 -minw 6 -maxw 50 -revcomp. Then, TOMTOM within the MEME suite was applied to test the similarity between the identified motifs and the binding motifs of known factors with the following parameters: -verbosity 1 -min-overlap 5 -dist pearson -evalue -thresh 10.

Chromatin regulator binding prediction

ChIP-seq peaks of all mouse chromatin regulators were downloaded from the Cistrome Data Browser(Zheng et al., 2019), and the transcription factors were displayed when they overlapped with the promoters (TSS ± 2k) of the 24 genes shown in Figure 4D. The GIGGLE score was calculated by the Cistrome DB Toolkit; chromatin regulators with values greater than 50 (the default confidence of the Cistrome DB Toolkit) were displayed, and redundancy was removed.

References

Bailey, T.L., Johnson, J., Grant, C.E., and Noble, W.S. (2015). The MEME Suite. Nucleic Acids Res 43, W39-49.

Gao, R., Wang, C., Gao, Y., Xiu, W., Chen, J., Kou, X., Zhao, Y., Liao, Y., Bai, D., Qiao, Z., et al. (2018). Inhibition of Aberrant DNA Re-methylation Improves Post-implantation Development of Somatic Cell Nuclear Transfer Embryos. Cell Stem Cell 23, 426-435 e425.

Huang, D.W., Sherman, B.T., and Lempicki, R.A. (2009). Systematic and integrative analysis of large gene lists using DAVID bioinformatics resources. Nat Protoc 4, 44-57.

Kim, D., Paggi, J.M., Park, C., Bennett, C., and Salzberg, S.L. (2019). Graph-based genome alignment and genotyping with HISAT2 and HISAT-genotype. Nat Biotechnol 37, 907-915.

Langmead, B., and Salzberg, S.L. (2012). Fast gapped-read alignment with Bowtie 2. Nat Methods 9, 357-359.

Li, H., Handsaker, B., Wysoker, A., Fennell, T., Ruan, J., Homer, N., Marth, G., Abecasis, G., Durbin, R., and Genome Project Data Processing, S. (2009). The Sequence Alignment/Map format and SAMtools. Bioinformatics 25, 2078-2079.

Pertea, M., Pertea, G.M., Antonescu, C.M., Chang, T.C., Mendell, J.T., and Salzberg, S.L. (2015). StringTie enables improved reconstruction of a transcriptome from RNA-seq reads. Nat Biotechnol 33, 290-295.

Zhang, Y., Liu, T., Meyer, C.A., Eeckhoute, J., Johnson, D.S., Bernstein, B.E., Nusbaum, C., Myers, R.M., Brown, M., Li, W., et al. (2008). Model-based analysis of ChIP-Seq (MACS). Genome Biol 9, R137.

Zheng, R., Wan, C., Mei, S., Qin, Q., Wu, Q., Sun, H., Chen, C.H., Brown, M., Zhang, X., Meyer, C.A., et al. (2019). Cistrome Data Browser: expanded datasets and new tools for gene regulatory analysis. Nucleic Acids Res 47, D729-D735.
